# The Role of Oral Pathobionts’ Outer Membrane Vesicles in Cancer Pathology and Therapeutic Development

**DOI:** 10.3390/cells15100855

**Published:** 2026-05-08

**Authors:** Sara Hadjigol, Bansari A. Shah, Negar Yazdani, Neil M. O’Brien-Simpson

**Affiliations:** ACTV Research Group, Division of Basic and Clinical Oral Sciences, Centre for Oral Health Research, Melbourne Dental School, Royal Dental Hospital, The University of Melbourne, Carlton, Victoria 3010, Australia; benny.shah@unimelb.edu.au (B.A.S.); negar.yazdani@student.unimelb.edu.au (N.Y.)

**Keywords:** oral microbiome, cancer, outer membrane vesicles, *Fusobacterium nucleatum*, *Porphyromonas gingivalis*, inflammation, microbial biomarkers, chemoresistance

## Abstract

**Highlights:**

This review highlights that outer membrane vesicles from oral bacteria disrupt mitochondrial function, promoting inflammation, metabolic reprogramming, and tumour progression.

**What are the main findings?**

- Oral bacterial OMVs contribute to cancer initiation, progression, and metastasis.
- OMVs from *P. gingivalis* and *F. nucleatum* promote chronic inflammation and immune evasion.
- OMVs disrupt mitochondrial dynamics, inducing ROS production and metabolic reprogramming.
- OMV-mediated signalling enhances tumour cell survival, invasion, and metastatic potential.
- Engineered OMVs show promise as platforms for cancer immunotherapy and drug delivery.

**What are the implications of the main findings?**

- Oral health is directly linked to cancer risk, poor oral hygiene and periodontal disease may actively fuel tumour initiation and progression, making dental care a potential cancer prevention strategy.
- A new mechanistic pathway to cancer has been identified, OMV-driven mitochondrial disruption and metabolic reprogramming represent a previously underappreciated route through which bacteria contribute to oncogenesis.
- Tumour metastasis may be partially bacterially driven, the ability of OMVs to enhance invasion and metastatic signalling implicates oral dysbiosis as a systemic, not just local, threat.
- Engineered OMVs open a new frontier in cancer therapy, their natural targeting and cargo-delivery properties make them compelling vehicles for immunotherapy and precision drug delivery, potentially with fewer side effects than synthetic nanoparticles.

**Abstract:**

Cancer remains one of the leading causes of mortality worldwide, with increasing recognition of the host microbiome as a modifiable contributor to tumour initiation and progression. Among microbial mediators, outer membrane vesicles (OMVs) derived from Gram-negative oral pathobionts have emerged as critical effectors of host–microbe interactions. These nanoscale vesicles function as delivery systems for a diverse range of bioactive cargo, including virulence factors, lipopolysaccharides, proteins, and nucleic acids, enabling both local and systemic modulation of host cellular processes. Emerging evidence suggests that OMVs produced by oral pathobionts, particularly *Porphyromonas gingivalis* and *Fusobacterium nucleatum*, are associated with tumour-promoting inflammation, immune dysregulation, epithelial transformation, and metastatic progression. Mechanistically, OMVs have been shown to activate key signalling pathways, disrupt mitochondrial function, induce oxidative stress, and reprogram the tumour microenvironment in ways that favour cancer cell survival and immune evasion. In addition, OMV-mediated modulation of host responses has been linked to resistance to anticancer therapies. In this review, we synthesize current evidence on the role of oral pathobionts’ OMVs in cancer biology, with a focus on their contributions to tumour initiation, progression, and metastasis. We further discuss emerging clinical associations, the potential of OMV-derived components as diagnostic biomarkers, and the growing interest in engineered OMVs as platforms for therapeutic intervention. Finally, we highlight key challenges and knowledge gaps that must be addressed to advance the translational application of OMV-based strategies in oncology. Overall, OMVs represent a promising but still evolving link between the oral microbiome and cancer, offering new insights into disease mechanisms and potential avenues for diagnosis and therapy.

## 1. Introduction

Cancer is the world’s second most common cause of death, responsible for almost 10 million fatalities annually. Developing and applying successful prevention and treatment methods is still challenging and requires a better understanding of the molecular mechanisms of cancer development and addressing manageable risk factors [1]. Cancer has traditionally been characterized as a genetic disorder, with many cases resulting from intrinsic, unmodifiable risks, such as the stochastic accumulation of mutations in rapidly dividing cell populations [2]. However, a significant portion of cancers and their progression can be attributed to modifiable risk factors, including tobacco use, alcohol consumption, high body mass index, and chronic infections [3]. Recently, research has focused on the host microbiome as a risk factor in the initiation and progression of tumours [4]. The acute inflammatory response is the body’s first line of defence against infection or injury, supporting both innate and adaptive immunity. However, if this response lingers and does not resolve, it can turn into chronic inflammation. This chronic state creates an immunosuppressive environment filled with cells like M2 macrophages, myeloid-derived suppressor cells (MDSCs), and regulatory T cells (Tregs), along with various regulatory cytokines [5,6]. These conditions have been linked to the activation of oncogenes, epigenetic alterations such as DNA methylation and histone modification, protein damage, and the release of reactive oxygen species (ROS), impacting multiple signalling pathways, such as nuclear factor-κB (NF-κB), K-RAS (involved in the regulation of cell division), and P53 (tumour suppressor), and potentially leading to the initiation and progression of cancer [7,8,9]. Concurrently, chronic local inflammation and its systemic effects have been recognized as foundational elements that support multiple hallmark functions of malignancies including proliferative signalling, evasion of growth suppressors, resistance to cell death, enabling of replicative immortality, induction of angiogenesis, and activation of invasion and metastasis [10].

In 1858, Rudolf Virchow was the first to observe a link between inflammatory responses and cancer development [11]. Over the following century, this observation laid the groundwork for the understanding that around 18% of human cancers originate from chronic inflammation [12]. The most well-known example of bacterial tumour induction is the development of gastric cancer caused by *Helicobacter pylori* through host–microbial interaction [13]. Additionally, *Schistosoma haematobium* is closely associated with bladder squamous cell carcinoma [14], and *Opisthorchis viverrini* is linked to cholangiocarcinoma [15]. Although these examples show a direct cause and effect relationship, in general the association between cancer and other bacterial infections and interactions is known to be more complicated. Interestingly, treatments aimed at reducing bacterial infections have been shown to improve the prognosis of cancers associated with these infections.

While certain bacteria, such as *H. pylori* and *Fusobacterium nucleatum*, are well-documented for their tumour-promoting roles, emerging evidence highlights the potential of other bacterial species that have anticancer effects. This duality suggests that microbial interactions with the host immune system and tumour microenvironment can either foster tumorigenesis or contribute to tumour suppression. Several bacterial strains, including *Salmonella typhimurium*, *Clostridium butyricum*, *Lactobacillus* spp., and *Streptococcus pyogenes*, have demonstrated promising anticancer properties through mechanisms such as immune modulation, induction of tumour cell apoptosis, and competition with pathogenic microbiota [16,17,18,19,20,21,22]. The relationship between the microbiota and cancer is complex and context-dependent, with certain microorganisms promoting tumorigenesis while others exhibit protective or anticancer effects. This dual role has been demonstrated in several animal models designed to investigate microbial modulation of tumour development [23,24,25]. For example, in murine models of colorectal cancer, administration of *Fusobacterium nucleatum* accelerates tumour growth and increases tumour burden through activation of pro-inflammatory signalling pathways and suppression of anti-tumour immune responses. Mechanistically, virulence factors such as the outer membrane adhesin Fap2 facilitate bacterial binding to tumour cells and interaction with immune inhibitory receptors, including TIGIT, thereby impairing NK and T cell activity. Furthermore, bacterial outer membrane vesicles (OMVs), which package and disseminate virulence factors and immunomodulatory molecules, may amplify these tumour-promoting effects within the tumour microenvironment.

Although associations have been identified between cancer pathology and oral or gut microbiota, identifying the specific bacterial species responsible in cancer initiation or progression is challenging due to the presence of over a million bacterial species in the human body. For instance, the colon harbours more than 100 trillion bacterial species, while the oral cavity contains over 500 different species. Additionally, a bacterial species implicated in cancer progression may employ varying mechanisms depending on the site [26]. The oral cavity serves as a significant microbiological reservoir within the human body, encompassing a diverse range of bacterial species, fungi, viruses, and bacteriophages. These complex multispecies communities generally sustain a balanced immunoinflammatory relationship with the host [27,28]. However, certain species, like the oral pathobionts *Porphyromonas gingivalis* (*P. gingivalis*), can disrupt this equilibrium, leading to a dysbiotic host–microbiota interaction. As a result, other community members, such as *Fusobacterium nucleatum* (*F. nucleatum*), can become opportunistically pathogens [29,30]. The combined effects of a dysbiotic microbial community and a dysregulated immune response ultimately cause periodontal disease. These extensively studied periodontal organisms have now become central to the emerging link between oral pathobionts and cancer.

A higher relative abundance of *Fusobacterium* in the stools of colorectal cancer patients was detected through 16S rRNA gene amplicon sequencing and biofilm quantification, indicating that microbial signatures may serve as non-invasive biomarkers [31]. Additionally, the presence of *F. nucleatum* in esophageal squamous cell carcinoma was validated through qPCR quantification of its DNA levels and was correlated with reduced survival rates [32]. Recent studies have shown that a high burden of *F. nucleatum* is associated with poor chemotherapeutic responses in two cohorts, suggesting that *F. nucleatum* may undermine the effectiveness of chemotherapy and enhance drug resistance in esophageal squamous cell carcinoma [33]. Due to its ability to disrupt epithelial tissues and host defence mechanisms, *P. gingivalis* is increasingly recognized for its potential role in tumour development [34]. Given that the orodigestive tract is a continuous, smooth passage, *P. gingivalis*, which exhibits significant mobility and invasion capabilities compared to other oral pathobionts, might disseminate throughout this area and potentially accelerate local tumorigenesis [35]. Additionally, the colonization and increase in *P. gingivalis* in the oral cavity has been linked to increased chemotherapeutic resistance, indicating that periodontitis may hinder the treatment of oral cancer [36]. Recent clinical research has supported a connection between oral pathobionts and esophageal cancer, with higher levels of *P. gingivalis* detected in subgingival plaque, saliva, and tumour tissues, correlating with an increased incidence of esophageal squamous cell carcinoma [37]. Epidemiological and mechanistic studies have linked *P. gingivalis* infection with an increased risk of pancreatic ductal adenocarcinoma (PDAC), as well as with tumour progression and poorer clinical outcomes. Elevated serum antibody levels against *P. gingivalis* have been reported in individuals who subsequently developed pancreatic cancer, suggesting prior exposure may be associated with disease risk. Moreover, higher antibody titres have been correlated with increased cancer-related mortality, supporting the potential utility of anti-*P. gingivalis* serology as a predictive or prognostic biomarker in cancer.

Although substantial evidence links oral pathobionts to cancer, the mechanisms by which the oral microbiome drives carcinogenesis through host-microbe interactions are yet to be fully elucidated. Emerging research suggests that outer membrane vesicles (OMVs) from oral pathobionts are critical mediators of these processes. OMVs encapsulate the virulence repertoire of their parent bacteria while exhibiting distinct nanoscale properties, including enhanced tissue penetration, immune modulation, and systemic dissemination, enabling them to influence host signalling independently of whole bacterial cells. While many studies report effects of bacterial infection, direct attribution to OMVs remains limited in some contexts. Consequently, this review adopts a framework whereby established mechanisms of oral bacteria-associated carcinogenesis are interpreted through an OMV-bias, as many of these effects are likely mediated or amplified by OMV-dependent processes. The initial sections therefore establish the biological context necessary to interpret this emerging field, while subsequent sections focus explicitly on the recent OMV-driven experimental, pathological, and translational evidence, defining their role as key effectors of cancer progression and potential therapeutic targets.

### 1.1. Bacterial Outer Membrane Vesicles

Outer Membrane Vesicles (OMVs) derived from Gram-negative bacteria are bilayered spherical nanostructures, typically ranging from 100 to 300 nanometers in size, with an internal cavity formed within the extracellular environment [38]. Although it was thought only Gram-negative bacteria produced membrane vesicles, recent work has shown that Gram-positive bacteria do produce membrane vesicle (MVs) and that different types of bacterial MVs besides OMVs are produced by bacteria not only by membrane blebbing, but are also formed by endolysin-triggered cell lysis [39]. For the purpose of this review we will focus on OMVs as the oral pathobionts associated with cancer are primarily Gram-negative. OMVs include those from oral pathobionts are composed of a phospholipid bilayer and contain lipopolysaccharides (LPS), membrane proteins, cell wall components, peptidoglycan, ion metabolites, signalling molecules, and nucleic acids [40,41].

**Figure 1:**
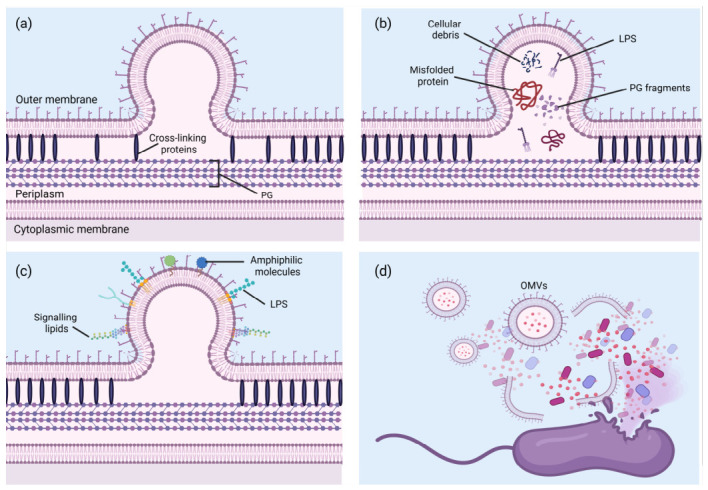
**Outer-Membrane Vesicle biogenesis mechanisms.** Multiple mechanisms are reported for the biogenesis of outer-membrane vesicles (OMVs). (**a**) Some cross-linking proteins provide the link between the outer phospholipid bilayer and the peptidoglycan layer. OMV production is increased in areas with disruption or loss of these cross-linking proteins. (**b**) Areas with the accumulation of peptidoglycan fragments, improperly folded proteins, or other cellular debris in the space between the outer phospholipid bilayer and the peptidoglycan layer generates internal pressure, which results in the bulging of the outer membrane and subsequent OMV production. (**c**) Charge balance, curvature and fluidity of the outer membrane can be altered following the insertion of foreign signal molecules (such as LPS, signalling lipids, or other amphiphilic molecules) and can create a favourable environment for OMV formation. (**d**) In cases of lysis, the cell membrane will be ruptured and released the fragments of the outer membrane and cytoplasmic/periplasmic components into the surrounding environment. These membrane fragments, rich in lipids and proteins, can spontaneously aggregate and self-assemble into bilayer vesicular structures, forming OMVs. Abbreviations: OMV: Outer membrane vesicle, LPS: Lipopolysaccharide, PG: peptidoglycan. (Figure created in BioRender.com, with permission).

The mechanisms underlying OMV generation include several distinct processes as shown in Figure 1 [42,43,44,45,46]. Briefly these processes are: (1) The disruption or loss of cross-linking proteins between the outer phospholipid bilayer and the peptidoglycan layer causes the outer membrane to gradually detach, leading to vesicle formation [47]. The bacterial cell envelope is structured to provide stability and integrity, with the outer membrane anchored to the underlying peptidoglycan layer by cross-linking proteins. These proteins, such as Braun’s lipoprotein in *Escherichia coli*, maintain the connection between the outer membrane and the rigid peptidoglycan. When these cross-linking proteins are disrupted or lost, the outer membrane begins to detach from the peptidoglycan layer. This detachment creates regions of instability where the membrane is no longer tightly secured, allowing it to bulge outward and eventually pinch off to form OMVs. This process is particularly significant during cell division or stress, where changes in the envelope structure can promote vesiculation. (2) The accumulation of excess peptidoglycan or misfolded proteins in the space between the outer phospholipid bilayer and the peptidoglycan layer generates internal pressure, which results in the bulging of the outer membrane and subsequent OMV production [48]. The space between the outer membrane and the peptidoglycan layer, known as the periplasm, can accumulate various materials such as excess peptidoglycan fragments, improperly folded proteins, or other cellular debris. This accumulation generates internal pressure within the periplasmic space. As the pressure increases, the outer membrane is forced to bulge outward, creating a protrusion that eventually pinches off as an OMV. This mechanism is particularly important under conditions of stress or during bacterial growth, where the synthesis of cell wall components and proteins may become unbalanced, leading to the buildup of materials that promote vesicle formation. Additionally, the formation of OMVs serves as a means for bacteria to dispose of unwanted or harmful cellular components, thus protecting the cell from damage. (3) The insertion of foreign signal molecules into the outer membrane alters its charge balance, promoting membrane curvature and facilitating OMV formation [49]. The outer membrane’s composition is crucial for maintaining the structural and functional integrity of Gram-negative bacteria. The insertion of foreign molecules, such as lipopolysaccharides (LPS), signalling lipids, or other amphiphilic molecules, can alter the charge balance and fluidity of the outer membrane. These changes can induce membrane curvature due to the asymmetric distribution of lipids or changes in electrostatic interactions. The bending of the membrane creates a favourable environment for OMV formation, as regions of high curvature can easily bud off as vesicles. This mechanism allows bacteria to respond to environmental signals or changes by adjusting their membrane composition, leading to the production of OMVs that can carry specific molecules, such as virulence factors, to target cells. (4) In cases of cell rupture and lysis, the loss of membrane integrity causes fragments of the disrupted plasma membrane to aggregate and self-assemble into OMVs [50]. In cases of extreme stress or cell death, bacterial cells may undergo lysis, leading to the rupture of the cell membrane. When this occurs, fragments of the outer membrane are released into the surrounding environment. These membrane fragments, rich in lipids and proteins, can spontaneously aggregate and self-assemble into vesicular structures, forming OMVs. This process is driven by the inherent biophysical properties of the membrane lipids, which tend to form bilayer structures in an aqueous environment. The formation of OMVs from membrane fragments during cell lysis serves as a last-resort mechanism for bacteria to release cellular components, potentially aiding in the dissemination of virulence factors or other molecules that can influence the surrounding microbial community or host environment.

### 1.2. Mechanisms of the Microbiota-Associated Carcinogenesis

There are several common mechanisms associated with microbiota-driven cancer development [51]. One mechanism is the colonization and survival of a cancer-associated microbiota. Prolonged colonization by pathogenic bacteria in this microbiota is known to facilitate carcinogenic effects [52]. The increase in the pathogen *F. nucleatum* in oral plaque is an example of this, *F. nucleatum* expresses several proteins, such as Fap2, that enable it to interact with both microorganisms and host cells [53,54], key to its ability to bind to and invade various cell types, including oral and colonic epithelial cells [55,56]. Fap2 specifically binds to an overexpressed membrane protein in colorectal cancer cells, unlike normal endocytosis mechanisms, leading to targeted and efficient invasion of these cells [57]. This unique binding process is problematic because, unlike normal endocytosis, it involves a specific interaction between *F. nucleatum*’s Fap2 protein and a receptor that is overexpressed on colorectal cancer cells. This interaction bypasses regular immune surveillance mechanisms, allowing the bacteria to selectively adhere to and invade tumour cells, rather than normal, healthy cells. As a result, it enhances tumour cell adhesion and invasion, contributing to the progression and metastasis of cancer. Additionally, the FadA adhesin of *F. nucleatum* binds to E-cadherin and activates the β-catenin signalling pathway, driving transcription of oncogenes and pro-inflammatory mediators that enhance tumour cell proliferation, survival, and metastasis. Persistent β-catenin activation disrupts epithelial homeostasis and promotes tumour progression, ultimately contributing to poorer clinical outcomes and therapeutic resistance [58]. Another periodontal pathogen *P. gingivalis*, known to increase in the oral microbiome in periodontal disease has been shown to survive in different acidic conditions affording its distribution and colonization of the upper digestive tract supporting the association with esophageal cancer [59].

A second mechanism involves changes in the microbial ecosystem leading to functional alterations. Oral diseases often stem from changes in microbial balance, where beneficial bacteria decrease, and harmful ones increase [60]. Inflammation and immunodeficiency are significant outcomes of oral microbiome dysbiosis which are also significant mechanisms in carcinogenesis [61]. Oral microbial dysbiosis has been linked to inflammation-associated colorectal cancer (CRC), highlighting the importance of the overall microbial ecosystem rather than just specific pathogens, although individual or a collection of pathogens may act as keystone organisms in this process [62]. A recent cross-cohort meta-analysis found greater oral microbial richness in colorectal cancer tissues, suggesting an influx of oral pathobionts [63]. In this study, the researchers isolated colorectal tissue samples from patients diagnosed with CRC and used polymerase chain reaction (PCR) analysis to identify specific oral pathogens. They discovered that oral pathobionts, including *F. nucleatum* and *P. gingivalis*, were present in the colorectal tissues, supporting the hypothesis that oral pathogens might contribute to CRC development. The study also examined the oral cavity and plaque of these patients and found a correlation between the presence of these pathogens in the oral cavity and their detection in CRC tissues.

Conversely, lower microbial richness and diversity were observed in salivary samples of patients with acute myelocytic leukemia compared to healthy controls [64]. Similarly, liver cancer patients showed increased microbial diversity in the tongue coat, while those with gastric cancer had reduced diversity in the tongue coat but increased diversity in salivary and subgingival samples [65]. Recently, it has been shown that salivary microbial diversity decreases with throat cancer progression [66]. In all of these studies, these findings suggest that changes in the oral microbiome from a healthy homeostatic state to some dysbiotic state have an impact and where and what these changes are is dependent on the cancer.

A third mechanism to consider is the ability of the oral microbiome to migrate to different parts of the body through different pathways such as blood circulation or orodigestive tract, which plays a significant role in the development of systemic cancers linked to oral infections and various other oral pathobionts’ diseases [67,68].

### 1.3. The Oral Microbiome’s Role in Carcinogenesis

Pathogenic oral pathobionts can cause various inflammatory diseases, and their carcinogenic effects are partly due to the inflammation they induce [69]. The imbalance in oral microbiome increases the production of inflammatory mediators like interleukin (IL-1) β, IL-6, and matrix metalloproteinases (MMPs), which are elevated in conditions like chronic periodontitis [70]. IL-1β promotes inflammation by releasing prostaglandins, tumour necrosis factor (TNF), and IL-6, and it also has the potential to enhance tumour metastasis [71,72]. IL-6 is involved in various stages of cancer development and is considered a therapeutic target in cancer treatment as IL-6 plays a critical role in driving chronic inflammation, which fosters a microenvironment conducive to carcinogenesis. Chronic inflammation is known to result in DNA damage, stimulate cellular proliferation, and inhibit apoptosis, thereby contributing to tumour initiation and progression [5,73]. A number of studies have shown that chronic inflammation is a major contributor to cancer onset and progression, e.g., inflammatory bowel diseases, such as ulcerative colitis and Crohn’s disease are known risk factors for developing colon cancer [74,75]. Further, many of the prognostic markers for cancer are inflammatory mediators, e.g., high mobility group box-1 (HMGB-1), CD-36, IL-11, Lipocalin 2 for prostate, oral, ovarian, breast and colon cancers and all of these are highly expressed in chronic periodontitis and specifically *P. gingivalis* [74,75,76,77,78,79,80].

The role of the oral microbiome in cancer is complex, as certain microorganisms promote tumour development whereas others may exert protective effects [10,81,82]. The current key mechanisms of how oral pathobionts have been implicated in cancer are summarized below, though further research is needed to gain deeper insights. Among tumour-promoting oral pathobionts, *P. gingivalis* has been strongly associated with chronic inflammation and oncogenic signalling in multiple malignancies, including pancreatic and oral squamous cell carcinoma [28,83,84]. While a moderate inflammatory response can protect against cancer, excessive inflammation is a major factor in cancer development [85]. Persistent infection with *P. gingivalis* induces sustained inflammatory responses characterized by increased production of pro-inflammatory cytokines, activation of NF-κB signalling, and modulation of cell survival pathways as described above [86,87,88]. Chronic inflammation is widely recognized as a hallmark of cancer and contributes to enhanced replicative potential, autonomous growth signalling, resistance to apoptosis, and increased angiogenesis [89,90,91]. In the context of oral dysbiosis, elevated levels of inflammatory mediators driven by *P. gingivalis* infection can promote epithelial transformation, tumour cell proliferation, and metastatic spread [82,92]. These inflammation-mediated alterations create a tumour-permissive microenvironment that supports cancer progression and therapeutic resistance [93].

Notably, *P. gingivalis* infection has been linked to changes in key oncogenic pathways. Microarray data analysis has shown an altered expression of genes such as IL-6, Stat1, and C-X-C motif chemokine ligand 10 (CXCL10) following *P. gingivalis* infection, these factors have been implicated as upstream regulators of carcinogenesis [66]. Increased levels of IL-8, resulting from *P. gingivalis* infection, is known to promote oral cancer by creating an inflammatory environment, facilitating tumour growth, and interacting with other carcinogenic processes [94,95]. *P. gingivalis* oral infection also results in the overexpression of MMPs and IL-1β, which are known to play a crucial role in tumour migration by degrading the extracellular matrix and disrupting cell adhesion [96].

Inflammation triggered by oral pathogens such as *P. gingivalis* and *F. nucleatum* is known to activate key pro-inflammatory transcription factors within epithelial and tumour cells, including NF-κB, Signal Transducer and Activator of Transcription 3 (STAT3), and Hypoxia-Inducible Factor 1α (HIF-1α) [97,98]. Activation of these pathways promotes the expression of critical cytokines and chemokines, such as TNF and IL-6 [73,99], as well as inflammatory enzymes including Cyclooxygenase (COX)-2 [100]. Through sustained bacterial stimulation, this signalling cascade establishes a chronic inflammatory state within the tumour microenvironment. The resulting chemokine gradients recruit host leukocytes, including macrophages, dendritic cells, mast cells, and T cells, to the tumour stroma to mediate the immune response [101]. Cytokine release within the tumour microenvironment further amplifies inflammation via autocrine and paracrine signalling [5,102], reinforcing tumour promoting pathways. Additionally, COX-2-mediated prostaglandin synthesis contributes to immune modulation, angiogenesis, and tumour cell survival [103,104,105]. Collectively, these pathogen-driven inflammatory networks create a tumour-permissive microenvironment that supports cancer progression and therapeutic resistance.

In the Janus kinase (Jak)/Stat3 pathway, IL-6 binding to its receptor activates JAK kinases, leading to the phosphorylation of Stat3. The phosphorylated Stat3 translocates to the nucleus, where it promotes the expression of genes that support cell survival, proliferation, angiogenesis, and metastasis [106]. Furthermore, IL-6 can modulate the immune response, impairing anti-tumour immunity by inhibiting cytotoxic T cell activity and promoting the expansion of Tregs and MDSCs [107]. At low but sustained levels, TNF induced by oral pathogens such as *P. gingivalis* and *F. nucleatum* may be insufficient to mediate effective anti-tumour immunity and instead contribute to a chronic inflammatory tumour microenvironment. Through direct bacterial interaction with tumour cells, or indirectly via paracrine signalling from infected stromal and immune cells, pathogen-driven TNF production can promote cell survival pathways, angiogenesis, and immune modulation, thereby facilitating tumour progression [99].

In vitro infection of oral squamous cell carcinoma (OSCC) cells and human gingival keratinocytes with *P. gingivalis* results in upregulation of the immune checkpoint receptors B7-H1 (PD-L1) and B7-DC (PD-L2), suggesting that bacterial exposure may facilitate immune evasion in oral cancers [108]. B7-H1, which is commonly expressed in many human cancers, plays a key role in helping tumour cells evade the immune system [109,110,111]. It induces the anergy and apoptosis of activated T cells, allowing cancer cells to overcome the host’s immune response [112]. Additionally, B7-H1 selectively triggers the production of IL-10 by antigen-presenting cells (APCs) during the priming of T lymphocytes, enhancing the immunosuppressive functions of APCs. *P. gingivalis* infection not only increases the expression of B7-H1 but also enhances the tolerance induced by APCs, leading to an upregulation of immunoglobulin-like transcript 3 (ILT-3) and B7-H1 expression [113]. This upregulation supports the development of Tregs, which are crucial for maintaining peripheral tolerance by actively suppressing effector T cells and preventing immune-mediated tissue damage [114]. Increased frequencies of immunosuppressive regulatory T cells (Tregs) have been reported in both the peripheral circulation and tumour microenvironment of patients with oral squamous cell carcinoma (OSCC) and numerous other cancers. Their accumulation suppresses anti-tumour effector T cell responses, facilitating immune evasion, tumour progression, and adverse prognosis [115,116]. B7-H1 also plays a role in modifying CD8^+^ T cell responses. In mouse models, B7-H1 expression in epidermal keratinocytes has been shown to reduce the function of cutaneous effector CD8^+^ T cells [117], while in liver sinusoidal endothelial cells, B7-H1-dependent signals modulate CD8^+^ T cell activity by affecting early IL-2 release [118]. Although the precise mechanisms remain incompletely defined, pro-inflammatory cytokines such as interferon-γ (IFNγ) are known to induce upregulation of B7-H1 (PD-L1), a key immune checkpoint molecule involved in tumour immune evasion. In oral Langerhans cells, activation of Toll-like receptor 4 (TLR4) increases B7-H1 expression in vitro [119], and in head and neck squamous cell carcinoma (HNSCC), elevated TLR4 expression correlates with higher tumour grade [120]. Importantly, oral pathogens may contribute to this immunosuppressive signalling axis. *P. gingivalis* activates TLR4-dependent inflammatory pathways, potentially enhancing PD-L1 expression within the tumour microenvironment [121]. Similarly, *F. nucleatum* has been shown to upregulate PD-L1 expression in cancer cells [122] and to promote tumour immune escape by impairing T cell-mediated cytotoxicity [123]. Collectively, these findings indicate that pathogen-driven modulation of PD-L1/B7-H1 signalling represents a critical mechanism by which oral pathobionts contribute to cancer pathogenesis, facilitating immune evasion and tumour progression.

Numerous pathogenic microbes contribute to the weakening of the host’s immune defence, with microbiota-associated immunosuppression playing a significant role in cancer formation. [124]. *F. nucleatum* is particularly influential in this process by shaping the immune microenvironment within tumours. This bacterium recruits immune cells to the tumour site, creating a proinflammatory milieu characterized by the accumulation of MDSCs and other immune cells that inhibit anti-tumour responses [6]. This environment is conducive to neoplastic development and growth [125]. A notable aspect of *F. nucleatum*’s impact is its ability to polarize macrophages towards the M2 phenotype, which is typically associated with anti-inflammatory responses and tissue repair. In the context of cancer, M2 macrophages can promote tumour growth and sustain a tumour-supportive environment. This M2 polarization is particularly evident in colorectal cancer associated with *F. nucleatum* infection in a toll-like receptor 4 (TLR4)-dependent manner, where it contributes significantly to the pro-tumour environment [126]. Moreover, *F. nucleatum* further undermines anti-tumour immunity by targeting natural killer cells and various T lymphocytes [127]. The bacterium achieves this through its Fap2 protein, which binds to specific receptors on these immune cells—known as T cell immunoreceptors with immunoglobulin and immunoreceptor tyrosine-based inhibitory motifs (TIGIT) domains. This interaction impairs the ability of NK cells and T lymphocytes to effectively mount an immune response against cancer cells, thereby facilitating tumour growth and survival.

In addition to the effects of *F. nucleatum*, *P. gingivalis* plays a critical role in cancer progression by promoting anti-apoptotic activity in cancer cells. The ability of cancer cells to resist apoptosis is a key factor contributing to their uncontrolled growth and resistance to chemotherapy. Research has shown that *P. gingivalis* can alter the intrinsic mitochondrial apoptotic pathway through the JAK1/AKT/STAT3 signalling cascade [128]. Specifically, *P. gingivalis* infection leads to the phosphorylation of BAD (Bcl-2-associated death promoter) and an increase in the ratio of the anti-apoptotic protein BCL2 (B-cell lymphoma 2) to the pro-apoptotic protein BAX (Bcl-2-associated X protein), significantly reducing the apoptotic activity in epithelial cells [129]. Additionally, *P. gingivalis* secretes an anti-apoptotic enzyme, nucleoside diphosphate kinase, which breaks down adenosine triphosphate (ATP) and inhibits the activation of P2X7 (P2X Purinoceptor 7) receptors, further decreasing apoptosis [130,131]. A study exposing human immortalized oral epithelial cells to *P. gingivalis* showed that prolonged and repetitive exposure led to cell morphological changes, increased proliferation, a higher S phase fraction in the cell cycle, and promoted cell migratory and invasive properties. This indicates that severe periodontitis might contribute substantially to oral carcinogenesis [132].

Certain oral pathobionts are known to exacerbate the production of Reactive Oxygen Species (ROS) and Reactive Nitrogen Species (RNS), thereby contributing to the carcinogenic process. Notable species include *Streptococcus* spp. (e.g., *S. oralis*, *S. mitis*, *S. sanguinis*, *S. gordonii*, *S. oligofermentans*) and *Lactobacillus* spp. (e.g., *L. fermentum*, *L. jensenii*, *L. acidophilus*, *L. minutus*), as well as Bifidobacterium adolescentis [133]. These microorganisms can create a pro-carcinogenic microenvironment in the oral cavity through sustained inflammation and the enhanced production of reactive species. These reactive species are byproducts of cellular metabolism, with their production significantly elevated during inflammatory responses triggered by microbial activity in the oral cavity [133,134]. ROS, generated during inflammatory processes as described above, have been extensively implicated in the pathogenesis of cancer. These reactive molecules contribute to carcinogenesis through several mechanisms [135]. (1) Cellular Transformation: ROS induce oxidative damage to DNA, leading to mutations, chromosomal instability, and subsequent cellular transformation. This oxidative stress, as described above, is a critical driver in the initiation of carcinogenesis. (2) ROS activate key survival pathways, such as NF-κB, which upregulates anti-apoptotic genes, thereby enhancing the survival of tumour cells under conditions that would normally induce apoptosis. (3) ROS facilitate tumour invasion and metastasis by increasing MMPs secretion, which degrade the extracellular matrix, and by promoting epithelial-to-mesenchymal transition, a process integral to metastasis. (4) ROS play a pivotal role in angiogenesis by stabilizing HIF-1α and inducing the expression of pro-angiogenic factors such as vascular endothelial growth factor. This process is essential for tumour growth and the metastatic spread of cancer cells. In addition to ROS-mediated signalling, emerging evidence suggests that OMV-induced mitochondrial dysfunction may also involve alterations in mitochondrial dynamics. In particular, dysregulation of dynamin-related protein 1 (DRP1), a key regulator of mitochondrial fission, has been associated with increased mitochondrial fragmentation and metabolic adaptation in cancer cells. Enhanced DRP1 activity promotes mitochondrial fission, which supports tumour cell survival under stress conditions.

Furthermore, OMV-induced ROS may contribute to stabilization of HIF-1α, a central regulator of cellular metabolism under hypoxic conditions. Stabilized HIF-1α promotes a shift towards aerobic glycolysis, a hallmark of cancer metabolism known as the Warburg effect. This metabolic reprogramming enables cancer cells to generate energy more rapidly and supports biosynthetic processes required for rapid proliferation [136,137].

RNS, including nitric oxide (NO) and its derivatives, also contribute to carcinogenesis through mechanisms analogous to those of ROS [138]. (1) RNS cause nitrosative stress, leading to DNA damage through base modifications, strand breaks, and the formation of mutagenic DNA adducts. These alterations contribute to genomic instability, a hallmark of cancer development. (2) RNS interact with and modulate key signalling pathways involved in cell proliferation, apoptosis, and immune responses, further promoting a tumorigenic environment.

Volatile sulphur compounds (VSCs), which are produced by specific Gram-negative oral pathobionts (e.g., *Porphyromonas gingivalis*, *Treponema denticola*, *Fusobacterium nucleatum*), exhibit significant toxic and pro-inflammatory effects [139]. The dual role of VSCs in toxicity and inflammation underscores their potential involvement in initiating and promoting carcinogenesis within the oral cavity, particularly when produced by periodontopathogenic species such as *P. gingivalis*, *T. denticola*, and *F. nucleatum*. Chronic inflammation induced by these compounds can drive the production of reactive oxygen and nitrogen species, further contributing to DNA damage, cellular mutations, and the disruption of normal cell signalling pathways. This pathogen-driven inflammatory environment fosters cellular transformation, survival, and proliferation, ultimately supporting the development and progression of cancer.

Gingipains, which are cysteine proteases produced by *P. gingivalis*, are key virulence factors implicated in various pathologies, including periodontitis and Alzheimer’s disease [140]. Recent evidence suggests that these proteases may also play a role in tumorigenesis. Gingipains have been shown to activate inflammatory signalling pathways and enhance the activity of MMP-9, potentially facilitating tumour migration and progression [141,142]. Additionally, various oral pathobionts, including those from the genera *Lactobacillus*, *Lactococcus*, *Bifidobacterium*, *Streptococcus*, *Leuconostoc*, and *Pediococcus*, generally considered to be ‘good’ bacteria and form part of the healthy oral microbiome, are known to produce lactic acid [143]. Research has identified a role for lactic acid in suppressing immune responses at cancer sites, indicating its potential as a target for therapeutic intervention in oncology [144,145]. However, the overproduction of lactic acid and other acidic metabolites can lead to a decrease in pH and create a hypoxic microenvironment that is highly conducive to tumour metastasis [146].

Lipopolysaccharide (LPS), a major pathogenic component of the outer membrane of Gram-negative bacteria, is ubiquitously present in many anaerobic oral microbiome. LPS is known for its potent ability to activate the host’s inflammatory response, a process that is increasingly recognized as a critical factor in the pathogenesis of inflammation-associated cancers. LPS triggers an inflammatory cascade by binding to TLR4 on immune cells, leading to the activation of NF-κB and subsequent upregulation of various pro-inflammatory cytokines [147]. Notably, LPS stimulation during oral infections significantly elevates the levels of cytokines such as IL-1β, IL-6, and TNFα, which are central to the inflammatory response and are involved in multiple aspects of cancer biology, including tumour promotion, progression, and metastasis [148]. LPS from *P. gingivalis* produces a relatively weak TLR4 response, in comparison to LPS from other Gram-negative bacteria; however, *P. gingivalis*-derived lipopeptides strongly activate TLR2, leading to immune dysregulation and enhanced inflammation. This exaggerated immune response contributes to a pro-tumorigenic microenvironment by sustaining chronic inflammation and disrupting normal immune surveillance [149,150,151].

### 1.4. Clinical Evidence and Translational Relevance of OMV

Significant clinical evidence has established a strong correlation between microbiota and carcinogenesis, advancing our understanding of the pathogenic mechanisms involved. However, further research is required to elucidate the fundamental biology of cancer-associated oral microbiome. In particular, greater insight is needed into how microbiota interact with host cells, influence other microorganisms, and shape the surrounding microenvironment. Notably, emerging evidence suggests that outer OMVs play a key role in mediating these interactions and may contribute to cancer progression. Emerging clinical evidence supports the association between oral pathobionts and cancer development, providing translational relevance to OMV-mediated mechanisms. Several studies have reported increased abundance of *F. nucleatum* in colorectal cancer tissues compared to adjacent normal tissues, with higher levels correlating with advanced disease stage, lymph node metastasis, and reduced overall survival [125,152,153,154]. Furthermore, *F. nucleatum* has been associated with resistance to chemotherapy and increased risk of tumour recurrence [155].

Similarly, *P. gingivalis* has been detected in various tumour types, including esophageal and pancreatic cancers, and is associated with increased tumour aggressiveness and poorer clinical outcomes [84,156]. Elevated antibody titres against *P. gingivalis* have also been linked to increased cancer risk, suggesting its potential role in disease prediction.

Although direct detection of OMVs in clinical samples remains limited, several studies have identified OMV-associated components, including LPS and bacterial virulence factors, in tumour tissues and circulation, supporting their potential systemic impact [30,157]. In addition, alterations in oral and gut microbiota profiles have been proposed as non-invasive biomarkers for cancer diagnosis and prognosis [158,159].

These findings collectively support the clinical relevance of oral pathobionts’ OMVs while highlighting the need for further studies specifically targeting OMV detection and characterization in patient-derived samples.

### 1.5. Overview of OMV-Mediated Mechanisms in Cancer

**Figure 2:**
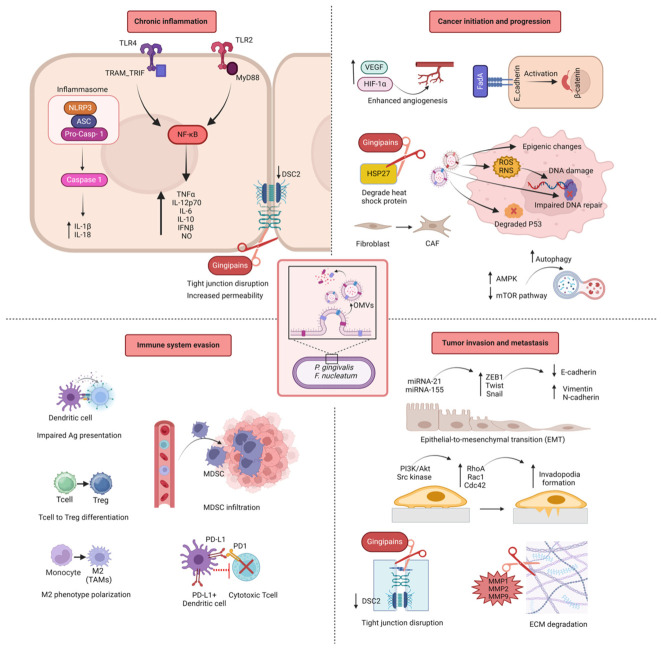
**OMV-mediated signalling pathways in cancer. from Oral Bacteria in cancer initiation, progression, and metastasis.** The cancer-promoting effects of derived OMVs are mediated by the virulence factors in their structure which provide the ability of OMVs to evade immune responses, cause chronic inflammatory conditions, induce mutations/cell proliferation, and enhance tumour invasion/metastasis. This happens through regulation of numerous molecular pathways resulting in the secretion of inflammatory cytokines, degradation of cellular junctions and ECM components, enhanced angiogenesis, higher cellular tolerance against stress and damages, impaired DNA repair, differentiation of immune cells to the ones involved in suppression of antitumor activity, formation of invadopodia, induction of EMT and activation of autophagy. TLR: Toll-like Receptor; TRAM: TRIF-related Adaptor Molecule; TRIF: TIR-domain-containing Adapter-inducing Interferon-β; MyD88: Myeloid Differentiation Primary Response 88; NLRP3: NOD-, LRR-, and Pyrin Domain-containing Protein 3; ASC: Apoptosis-associated Speck-like Protein Containing a CARD; NF-κB: Nuclear Factor kappa-light-chain-enhancer of Activated B Cells; IL: Interleukin; TNF: Tumour Necrosis Factor; IFN: Interferon; DSC: Desmocollin; VEGF: Vascular Endothelial Growth Factor; HIF: Hypoxia-Inducible Factor; FadA: Fusobacterium Adhesin A; HSP: Heat Shock Protein; CAF: Cancer-Associated Fibroblast; ROS: Reactive Oxygen Species; RNS: Reactive Nitrogen Species; AMPK: AMP-activated Protein Kinase; mTOR: Mechanistic Target of Rapamycin; TAM: Tumour-associated Macrophage; PD: Programmed Death; PD-L: Programmed Death-Ligand; ZEB1: Zinc Finger E-box Binding Homeobox 1; PI3K/Akt: Phosphoinositide 3-kinase/Protein Kinase B; Src: Proto-oncogene Tyrosine-protein Kinase Src; MMP: Matrix Metalloproteinase; ECM: Extracellular Matrix. (Figure created in BioRender.com).

In the context of cancer, OMVs have recently gained attention for their ability to manipulate host cellular processes, including the promotion of tumorigenesis, progression, and metastasis. OMVs are small membrane particles released by bacteria, which can carry a range of biologically active molecules such as proteins, lipids, and nucleic acids that influence the host microenvironment. This section provides an in-depth look at how OMVs from oral pathobionts, particularly Gram-negative *P. gingivalis* and *F. nucleatum*, influence each stage of cancer development, from initiation to metastasis (Figure 2). *P. gingivalis*, a key periodontopathic bacterium, has been implicated in promoting inflammation and immune modulation within the tumour microenvironment [160]. Studies suggest that *P. gingivalis* OMVs can facilitate tumour initiation and progression by promoting epithelial-to-mesenchymal transition (EMT), as described above, enhancing cancer cell survival, and modulating the immune response in favour of tumour growth [161]. On the other hand, *F. nucleatum*, which has been frequently found in various cancers such as colorectal cancer, also releases OMVs that contribute to cancer cell adhesion, invasion, and metastatic potential [162]. *F. nucleatum* OMVs are thought to interact with host cells by triggering the activation of pro-inflammatory signalling pathways, which can ultimately create a more favourable environment for tumour progression and metastasis [163]. Together, these bacteria, through their OMVs, play a significant role in altering the cancer microenvironment, influencing the various stages of cancer development and providing insight into the potential therapeutic implications of targeting OMVs in cancer treatment [164].

While both *P. gingivalis* and *F. nucleatum* contribute to cancer-associated processes through OMV-mediated mechanisms, their modes of action exhibit important mechanistic differences (Table 1). OMVs derived from *P. gingivalis* are characterized by the presence of proteolytic enzymes such as gingipains, which play a central role in host protein degradation, immune modulation, and disruption of epithelial barrier integrity [165,166]. These OMVs preferentially engage Toll-like receptor 2 (TLR2)-dependent signalling pathways and are associated with immune evasion mechanisms, including the upregulation of PD-L1 expression, impairment of dendritic cell maturation, and induction of regulatory T cells [88,167]. In addition, *P. gingivalis* OMVs have been linked to mitochondrial dysfunction, oxidative stress, and anti-apoptotic signalling, thereby promoting tumour cell survival [128,132]. 

In contrast, OMVs from *F. nucleatum* primarily exert their oncogenic effects through adhesins such as FadA and Fap2. FadA-mediated binding to E-cadherin activates β-catenin signalling, driving epithelial cell proliferation and oncogene expression [58], while Fap2 facilitates immune evasion through interaction with the TIGIT receptor on immune cells [171]. *F. nucleatum* OMVs predominantly activate TLR4-dependent inflammatory pathways and are associated with modulation of the tumour immune microenvironment, including recruitment of myeloid-derived suppressor cells and macrophage polarization [125,170].

These distinctions highlight that, although both species contribute to tumour-promoting processes, *P. gingivalis* OMVs are more closely associated with protease-driven immune modulation and metabolic reprogramming, whereas *F. nucleatum* OMVs primarily promote tumour progression through adhesion-mediated signalling and immune suppression pathways.

### 1.6. OMVs in Cancer Initiation

OMVs released by *P. gingivalis* and *F. nucleatum* are known to play crucial roles in cancer initiation [36,179]. These OMVs contain various pathobiont-associated molecular patterns (PAMPs) of bacterial cell components, such as lipopolysaccharide (LPS), deoxyribonucleic acid (DNA), ribonucleic acid (RNA), peptidoglycan (PGN), outer membrane proteins, enzymes, and toxins that can interact with host cells and modulate key processes such as inflammation, immune response, DNA damage, and the tumour microenvironment (TME) [41,180,181,182]. Both *P. gingivalis* and *F. nucleatum* have been associated with cancers like oral squamous cell carcinoma (OSCC) and colorectal cancer (CRC), respectively. This section discusses their shared and distinct mechanisms in promoting cancer initiation.

Chronic inflammation is a well-established driver of cancer initiation, and OMVs from *P. gingivalis* and *F. nucleatum* are central players in sustaining this inflammatory environment. OMVs from both *P. gingivalis* and *F. nucleatum* activate Toll-like receptors (TLRs) on host immune and epithelial cells, triggering inflammatory signalling pathways. OMVs from *P. gingivalis* contain LPS, gingipains (proteases), and other virulence factors that engage TLR2 and TLR4 receptors on epithelial cells and immune cells [183]. This leads to activation of nuclear factor-κB (NF-κB) and mitogen-activated protein kinase (MAPK) pathways, resulting in the production of pro-inflammatory cytokines like IL-6, TNFα, and IL-1β [184]. Similarly, *F. nucleatum* OMVs also contain LPS and antigenic components with potential virulence functions (such as FomA, FadA, FadD, Fad-I, NapA, ClpB, GroEL, TraT and YadA) triggering TLRs on epithelial or immune cells and enhancing the inflammatory response by promoting NF-κB and increasing proinflammatory cytokines secretion (e.g., TNFα and IL-8) to initiate inflammation [179,185]. It has been specifically indicated that *F. nucleatum* derived OMVs (from polymorphum subsp.) can activate TLR4 and NF-κB pathway and consequently increase IL-8 (eight-fold) and TNF- α (six-fold) in human colon cell line HT-29 [185]. Chronic exposure to inflammatory cytokines (IL-6, IL-1β, TNFα) produced by TLR activation creates a pro-inflammatory environment sustaining tissue damage and immune cell infiltration which proceed to a tumour microenvironment and increases the risk of cancer initiation [153]. Studies have also reported that increased TLR-4 expression correlates positively with tumour grade in head and neck squamous cell carcinoma (HNSCC).

The adhesive protein FadA is a mediator for the pathogenicity of *F. nucleatum* [186,187]. It is reported that FadA activates oncogenic Wnt/β-catenin pathway through its binding to VE-cadherin on endothelial cells and to E-cadherins on epithelial cells [58,179]. Thereby, *F. nucleatum*-derived OMVs can initiate inflammatory and carcinogenic reactions by containing FadA in their structure. A recent study suggests that *P. gingivalis* OMVs due to having PAMPs like LPS, gingipains, and fimbriae in their structure, could directly stimulate immune evasion, inflammation, proliferation and invasion/migration in cancer cells. *P. gingivalis* OMVs activate TLR4 and thereby increase the expression of programmed death ligand (PD1/PD-L1) in T cells. It is known that the simultaneous expression of T cell exhaustion by PD1 and an increase in PD-L1 results in rapid progression of gastric tumours and a decrease in patient survival [188,189]. Similarly, PGN delivered within *P. gingivalis*-derived OMVs has shown the ability to trigger cytosolic NOD receptors to induce PD-L1 expression through a receptor-interacting protein kinase 2 (RIP2)-dependent mechanism in prostate and oral cancer cells [167,190].

### 1.7. OMVs in Cancer Progression

Purified *P. gingivalis* OMVs have been found to strongly activate the NF-kB pathway and production of proinflammatory cytokines (TNFα, IL-12p70, IL-6, IL-10, IFNβ, IL-8, IL-1b and NO), induce inflammasome activation (through activating Caspase-1 and producing IL-18), and trigger pyroptosis in macrophages [191,192]. These immune responses have been associated with increased cancer pathology, contributing to tumour progression and immune evasion. In addition, LPS, *P. gingivalis*-OMVs have been reported to be responsible for increasing proinflammatory cytokines, thereby maintaining chronic inflammation.

Two studies reported that chronic inflammatory conditions can modify the tumour microenvironment and contribute to tumour immune evasion in gastric cancer (GC) and prostate cancer [167,188]. Specifically, Muñoz-Medel et al. [188] highlighted how *P. gingivalis* plays a crucial role in fostering an inflammatory environment that may promote immune evasion in GC. This includes the alteration of immune cell infiltration and the upregulation of immune checkpoint proteins, which may enable cancer cells to escape immune surveillance. Similarly, it is demonstrated that *P. gingivalis* can upregulate PD-L1 expression in prostate cancer cells, further supporting the idea that chronic inflammation caused by this bacterium can enhance immune suppression within the tumour microenvironment [190]. These findings underscore the potential link between chronic infection in the oral cavity, inflammation, and tumour immune evasion, which may contribute to cancer progression.

Continuous inflammatory signalling driven by OMVs promotes the generation of ROS and Reactive Nitrogen Species (RNS) [138]. These reactive molecules cause DNA damage, such as single-strand breaks and base modifications (e.g., 8-oxoG), which, if not properly repaired, can lead to mutations and genomic instability, key events in cancer initiation. The oxidative stress generated by inflammation results in significant DNA damage. *P. gingivalis* OMVs enhance the production of ROS, leading to oxidative DNA damage in epithelial cells [193]. Additionally, OMVs can modulate host cell DNA repair pathways, inhibiting the proper repair of damaged DNA, thereby increasing the risk of mutations and chromosomal aberrations. *F. nucleatum* has been associated with colorectal cancer subtypes marked by high levels of microsatellite instability (MSI) [125]. OMVs from *F. nucleatum* can impair DNA repair mechanisms such as the mismatch repair (MMR) system, leading to the accumulation of mutations that drive cancer initiation. OMVs from both bacteria can interfere with the host’s DNA repair processes, further promoting genomic instability. Gingipains, proteases found in *P. gingivalis* OMVs, degrade key tumour suppressor proteins like p53, impairing the cell’s ability to respond to DNA damage. This loss of tumour suppressor function promotes unchecked cell proliferation and increases the risk of oncogenic mutations.

In addition to direct DNA damage, OMVs from *P. gingivalis* can induce epigenetic changes in host cells, such as DNA methylation and histone modifications, that silence tumour suppressor genes or activate oncogenes. For example, *P. gingivalis* OMVs have been linked to the hypermethylation of p16, a key tumour suppressor gene, thereby contributing to uncontrolled cell proliferation. *F. nucleatum* OMVs can also disrupt the DNA damage response, resulting in impaired repair of double-strand breaks and other DNA lesions, which are critical for maintaining genomic integrity in normal cells. OMVs from *P. gingivalis* and *F. nucleatum* can directly interact with host epithelial cells, leading to altered cellular signalling, DNA damage, and other pro-tumorigenic changes. *F. nucleatum* OMVs contain adhesins, such as FadA, which bind to E-cadherin on the surface of epithelial cells [186], which activates β-catenin signalling, a pathway known to promote cell proliferation and survival, thus contributing to cancer initiation [58].

Both *P. gingivalis* and *F. nucleatum* OMVs modulate host immune responses in ways that promote immune evasion and create a permissive environment for early-stage cancer cells to proliferate [194]. To highlight the mechanistic differences between major oral pathobionts, a comparative summary is provided in Table 1. OMVs from both bacteria can impair dendritic cell maturation and antigen presentation, which compromises the host’s ability to mount effective anti-tumour immune responses. OMVs from *P. gingivalis* reduce the ability of dendritic cells to mature and present antigens to cytotoxic T cells. This weakens the immune system’s ability to recognize and destroy pre-cancerous or cancerous cells. OMVs from *F. nucleatum* promote the differentiation of T cells into regulatory T cells (Tregs), which suppress anti-tumour immunity. By increasing the presence of Tregs in the tumour microenvironment, *F. nucleatum* OMVs create an immunosuppressive milieu that allows cancer cells to escape immune surveillance. Both *P. gingivalis* and *F. nucleatum* OMVs promote the polarization of macrophages into the M2 phenotype (tumour-associated macrophages or TAMs), which support tumour growth. TAMs secrete anti-inflammatory cytokines like IL-10 and TGF-β, which suppress cytotoxic immune responses. In addition, TAMs promote tissue remodelling and angiogenesis, both of which are critical for supporting early tumour growth and metastasis.

OMVs from *P. gingivalis* and *F. nucleatum* contribute to remodelling the tumour microenvironment by influencing stromal cells, immune cells, and the extracellular matrix, creating conditions that support the initiation and progression of cancer [125,195,196,197]. OMVs recruit immune cells that contribute to chronic inflammation. *F. nucleatum*, for example, is known to promote the infiltration of myeloid-derived suppressor cells (MDSCs) into tumours. These cells suppress anti-tumour immunity while producing pro-inflammatory factors that promote tumour growth. OMVs from both bacteria can activate fibroblasts and transform them into cancer-associated fibroblasts (CAFs) through pathways like TGF-β. CAFs play a vital role in creating a supportive niche for early-stage cancer cells by secreting growth factors, extracellular matrix (ECM) components, and cytokines that promote cell proliferation and survival. Gingipains in *P. gingivalis* OMVs degrade ECM components and epithelial cell junctions, allowing pre-cancerous or cancerous cells to invade surrounding tissues and establish a tumour.

Both *P. gingivalis* and *F. nucleatum* OMVs promote angiogenesis—the formation of new blood vessels—which is essential for tumour growth. OMVs from these oral pathobionts induce the production of vascular endothelial growth factor (VEGF) in stromal and epithelial cells. VEGF stimulates the formation of new blood vessels, providing oxygen and nutrients to rapidly proliferating tumour cells. OMVs from both bacteria can activate hypoxia-inducible factor 1-alpha (HIF-1α), even under normoxic conditions. This leads to the upregulation of angiogenic factors that enhance the blood supply to early tumours.

The hallmark of tumours lies in their ability to sustain continuous growth signals, enabling unchecked cell proliferation [10]. Concurrently, local invasion is a pivotal step in the invasion-metastasis cascade, marking the initiation of tumour spread and serving as a critical event in the progression of malignant tumours. Recent studies have highlighted that OMVs can significantly affect the regulation of tumour cell growth signals. Moreover, OMVs not only contribute to carcinogenesis but also enhance the local invasiveness of tumours, thus playing a crucial role in facilitating tumour progression and metastasis [198].

OMVs have been implicated in the activation of autophagy, a vital cellular recycling mechanism that cancer cells exploit to endure hostile conditions such as hypoxia, nutrient deprivation, and chemotherapeutic stress [199]. By inducing autophagy, OMVs enhance cancer cell survival and foster tumour growth within the harsh tumour microenvironment [200]. OMVs downregulate the mTOR (mechanistic target of rapamycin) signalling pathway, a key regulator that normally inhibits autophagy [201]. Suppression of the mTOR pathway, either by direct OMV interactions with mTOR or through the activation of upstream regulators such as AMP-activated protein kinase (AMPK), promotes autophagic activity [202]. This autophagic response helps cancer cells maintain metabolic homeostasis and survive despite the metabolic stress imposed by the tumour microenvironment. The autophagy triggered by OMVs also provides cancer cells with resistance to apoptosis, a form of programmed cell death [203]. By degrading damaged organelles and misfolded proteins that could otherwise lead to cell death, autophagy allows cancer cells to avoid apoptosis, further enhancing their survival and promoting tumour progression [41]. It is noteworthy that gingipains in *P. gingivalis*-derived OMVs are able to degrade 27 kDa heat shock protein (HSP27). HSP27 is a chaperone that responds to stress and apoptosis; the reduction of which, causes impairment in cell response to stress signals and their protection against damage, and in GC this has been shown to lead to cancer progression [188,204]. Another component of *P. gingivalis* derived OMVs, Mfa1 and FimA fimbriae, also upregulates C-X-C chemokine receptor type 4 (CXCR4) and downregulates Forkhead Box O1/3 (FOXO1/3), which activate antiapoptotic pathways and result in enhanced tumour progression [205].

### 1.8. OMVs in Metastasis

Metastasis is a critical process in cancer progression, where cancer cells disseminate from the primary tumour to distant organs, establishing secondary tumours [206]. OMVs significantly contribute to this process by altering the tumour microenvironment, enhancing cancer cell migration, and facilitating the formation of pre-metastatic niches, thereby promoting metastasis (Table 2). One of the critical ways OMVs influence cancer development is by enhancing the metastatic potential of cancer cells through the promotion of epithelial-to-mesenchymal transition (EMT). In this process, epithelial cancer cells lose their adhesive properties and gain mesenchymal characteristics, such as increased motility and invasiveness [207,208]. This transformation is essential for tumour cells to spread from the primary tumour site and invade distant tissues. OMVs, particularly through components like LPS, activate TLRs on cancer cells. This activation triggers key transcription factors such as Snail, Twist, and ZEB1, downregulating epithelial markers (e.g., E-cadherin) and upregulating mesenchymal markers (e.g., N-cadherin, vimentin) [209]. This phenotypic switch enhances the cancer cell’s ability to migrate and invade surrounding tissues, a hallmark of metastatic progression. OMV-associated miRNAs, such as miR-21 and miR-155, are known to be involved in the regulation of genes that control EMT [210,211]. These miRNAs can suppress the expression of tumour suppressors and promote the expression of transcription factors like Snail, Twist, and ZEB1, which are master regulators of EMT [212]. The induction of EMT by bacterial OMVs thus has significant implications for the progression of cancer, particularly metastasis.

In 2023, Chen et al. reported the role of *F. nucleatum* OMVs in promoting EMT induction in cancer cells. This transition is a result of changes in protein homoeostasis (downregulation of E-cadherin, and upregulation of Vimentin and N-cadherin) and thereby activating autophagy [176]. Investigating the HNSCC metastasis, CAL27/HSC3 cell migration and invasion has shown to be promoted by the time and concentration dependent presence of *F. nucleatum* OMVs due to the EMT-related changes which is reported to be linked with increased HNSCC tumour metastasis [220]. It is noteworthy that no promotion in oral cancer cells migration and invasion has been observed in the presence of *Escherichia coli*, *Lactobacillus rhamnosus* and *Streptococcus salivarius* derived OMVs [176].

Notably, OMVs derived from non-pathobiont or commensal bacteria, such as *Escherichia coli*, *Lactobacillus rhamnosus*, and *Streptococcus salivarius*, do not exhibit comparable pro-tumorigenic effects. This difference is primarily attributed to variations in OMV cargo composition and immunogenic properties [42,168]. OMVs from oral pathobionts are enriched with virulence factors, including proteases, adhesins, and immunomodulatory molecules, which actively manipulate host signalling pathways and promote tumour-supportive conditions. In contrast, OMVs derived from commensal bacteria typically lack these virulence-associated components and may instead contribute to immune homeostasis or exhibit neutral or even protective effects within the host [42,168]. Furthermore, differences in receptor engagement may also underlie these contrasting outcomes. Pathobiont-derived OMVs often trigger sustained activation of pro-inflammatory and oncogenic pathways, whereas commensal-derived OMVs tend to induce more regulated or transient immune responses. These findings highlight the importance of OMV origin and cargo composition in determining their functional impact on cancer-related processes.

Emerging evidence also reveals that *P. gingivalis* derived OMVs consists of different packaged small RNAs (sRNAs) is potential for targeting the function and/or stability of host mRNAs. A study conducted by Liu et al. indicated that sRNA23392 in *P. gingivalis* OMVs can decrease the expression of Desmocollin-2 (DSC2) in OSCC cells [221]. DSC2, a type of desmosomes, is an important junctional complex in epithelial and some non-epithelial tissues providing intercellular adhesion and is considered as a reliable tumour marker, particularly for epithelial tumours [222]. Downregulation of DSC2 is known to be correlated with the reduction in cell–cell adhesion, redistribution of adherence junctions and promotion of cell aggressiveness which represents shorter patient survival, higher tumour grading, and positive lymph node status in various cancer types such as pancreatic ductal adenocarcinoma, oral cancer, esophageal squamous cell carcinoma, colorectal carcinoma [223,224,225]. Gingipains in *P. gingivalis*-OMVs enhance tumour invasion and metastasis by degrading tight junction components between the gingival and the gastrointestinal epithelia, thus compromising their integrity [226]. It is shown that gingipains specifically cleave critical tight junction proteins such as occludin and claudins, which are essential for epithelial cell–cell adhesion and barrier function. This disruption of tight junctions facilitates the detachment of epithelial cells, enabling cancer cells to invade surrounding tissues and metastasize. Additionally, these modifications in epithelial integrity activate downstream signalling pathways that promote cellular motility and invasion, further supporting the progression of tumours. The remodelling of the cytoskeleton is a crucial process for cancer cell migration and invasion, and OMVs have been identified as important agents in influencing this reorganization [227]. OMVs have been shown to induce the rearrangement of the actin cytoskeleton, a dynamic network of actin filaments essential for cell shape, motility, and interaction with the extracellular matrix (ECM), facilitating the invasion of surrounding tissues, and ultimately promoting cancer metastasis. OMVs can activate Rho GTPases, which are molecular switches that regulate the organization of actin filaments. Rho family members such as RhoA, Rac1, and Cdc42 play critical roles in cytoskeletal remodelling by controlling the polymerization and depolymerization of actin filaments, facilitating the formation of invadopodia [228]. Additionally, OMVs stimulate signalling pathways such as the PI3K/Akt and Src kinase pathways, both of which are involved in invadopodia formation and cancer cell invasion [229]. OMVs also increase the secretion of matrix metalloproteinases (MMPs), particularly MMP-2 and MMP-9, which are responsible for degrading ECM components, e.g., collagen [230]. The degradation of ECM components allows tumour cells to break through tissue barriers and invade surrounding tissues, thus promoting cancer progression [231]. For example, OMVs from *P. gingivalis* have been demonstrated to disrupt the actin cytoskeleton and induce invadopodia formation in epithelial cells, which increases cancer cell invasion and contributes to metastasis [232]. Moreover, a study about gastric cancer (GC) reported that the LPS and gingipains in *P. gingivalis* derived OMVs play a role in activation of MMPs (MMP1, 2, and 9), through protease-activated receptor 2 (PAR2) and the ERK1/2-Ets1/3-p38 pathways, which results in enhanced tumour invasion and metastatic capabilities in GC [188,233].

**Figure 3:**
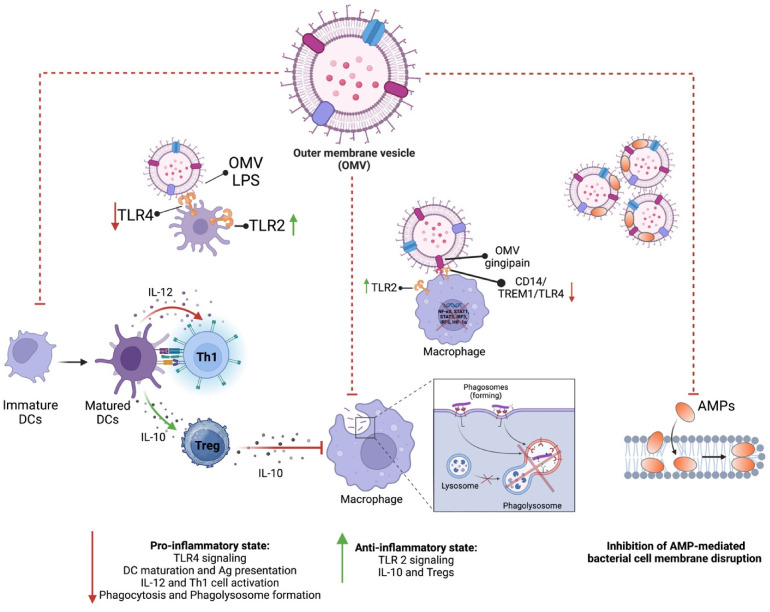
**Differential TLR signalling induced by OMVs.** Immune evasion strategies employed by OMVs suppress the host immune response, promoting bacterial survival and chronic infection. OMV-derived LPS preferentially activates TLR2 while inhibiting TLR4 signalling, resulting in an anti-inflammatory state characterized by IL-10 production and regulatory T cell (Treg) activation. In contrast, TLR4 signalling would typically induce a pro-inflammatory response through IL-12 production and Th1 cell activation. Dendritic cell (DC) maturation, antigen (Ag)-presentation, and T cell activation are also impaired. OMV-associated gingipains degrade or inhibit macrophage receptors CD14 and TREM/TLR4, thereby blocking downstream pro-inflammatory pathways. OMVs also disrupt phagolysosome formation in macrophages, preventing bacterial degradation and immune clearance. Furthermore, OMVs inhibit the activity of antimicrobial peptides (AMPs), which normally disrupt bacterial cell membrane, thus further promoting bacterial survival within the host. Induction of a pro-inflammatory or anti-inflammatory state is indicated by red downward arrows or green upward arrows, respectively.

OMVs contribute to immune evasion, a critical factor in cancer progression, by manipulating the host immune response and enabling tumour cells to evade immune surveillance (Figure 3) [234]. This immune escape mechanism allows tumour cells to proliferate and metastasize unchecked. OMVs have also been shown to impair the function of dendritic cells, which are critical for initiating anti-tumour immune responses. By disrupting the ability of dendritic cells to present antigens and activate T cells, OMVs contribute to the tumour’s ability to evade immune detection and destruction, enabling continued tumour growth and progression. Based on the proteome of *F. nucleatum* derived OMVs, it has been reported that 6 out of 98 proteins were autotransporter proteins, one of which is Fap2 protein [235]. Fap2 plays a key role in protecting colorectal cancer (CRC) cells and promoting tumour progression by modulating the host’s immune response [236]. It is shown that Fap2 achieves this immune evasion by directly interacting with key immune components [127,237]. It inhibits natural killer (NK) cell cytotoxicity, reduces the activity of tumour-infiltrating lymphocytes, and prevents T cell-mediated attacks on tumour cells. Specifically, Fap2 binds to the T cell immunoglobulin and ITIM (TIGIT) receptor domain, which is an inhibitory receptor on immune cells, effectively dampening the immune response. This immune suppression allows CRC cells to escape immune surveillance, creating an environment conducive to tumour growth and progression. By restraining the immune system’s ability to target cancer cells, Fap2 contributes significantly to the persistence and advancement of CRC. The binding of LPS in *F. nucleatum* derived OMVs to TLR-4 in tumour cells resulted in an increase in cell proliferation and secretion of several cytokines. Moreover, triggered TLR4 enables tumour cells to be protected from lysis mediated by human natural killer 92 (NK-92) cells and its ligation on tumour cells causes tumour progression [120].

Outer membrane vesicles (OMVs) derived from oral pathobionts such as *Porphyromonas gingivalis* and *Fusobacterium nucleatum* enhance tumour cell plasticity, thereby promoting more aggressive cancer phenotypes. These nanoscale vesicles are enriched with bioactive molecules—including proteins, lipids, lipopolysaccharide (LPS), and nucleic acids- that can directly reprogram tumour cell signalling. For example, OMVs can deliver virulence factors such as gingipains and LPS into the tumour microenvironment, activating pathways associated with epithelial–mesenchymal transition (EMT). EMT enables epithelial cells to acquire mesenchymal characteristics, including enhanced motility, invasiveness, and resistance to apoptosis, which are hallmarks of aggressive tumour behaviour [177]. In addition, OMVs can modulate immune signalling within the tumour microenvironment, altering interactions between tumour cells, stromal components, and immune cells, thereby increasing tumorigenicity and metastatic potential [238]. This OMV-driven enhancement of tumour plasticity allows cancer cells to adapt to dynamic microenvironmental pressures, evade immune surveillance, and resist therapeutic interventions, ultimately contributing to poorer clinical outcomes.

OMVs can promote cancer cell proliferation by delivering factors including small RNAs, proteins, LPS, sphingolipids (SL), flagellin, and other cargos that can stimulate oncogenic signalling pathways such as PI3K/Akt, MAPK, and Wnt, leading to increased cell survival, growth, and proliferation [198,239,240]. For instance, *P. gingivalis* OMVs have been shown to deliver gingipains, that activate oncogenic pathways like NF-κB and PI3K/Akt in oral cancer cells, leading to enhanced proliferation [241]. The persistent inflammation caused by these bacterial OMVs also promotes a tumour-supportive environment in the oral cavity.

The abovementioned mechanisms of impact of oral-bacterial derived OMVs in inducing the development, progression, and metastasis of cancers both in in situ and distal areas highlights the importance of further studies required in the field to assess the impact of OMVs originated from various oral pathobionts species on different cancer types. A summary of key OMV-mediated mechanisms and supporting evidence is presented in Table 2.

### 1.9. Outer Membrane Vesicles-Mitochondria Crosstalk

While much attention has been focused on the OMVs’ role in immune evasion and inflammation, recent evidence highlights that they are highly interactive with mitochondria—the cellular organelles responsible for energy metabolism and regulation of cell death. Mitochondria are not only essential for energy production through oxidative phosphorylation but also act as signalling hubs that modulate immune responses, apoptosis, and autophagy [242]. The emerging concept of OMV–mitochondria interaction suggests a critical role in bacterial pathogenesis and immune modulation. Understanding these interactions provides new insights into mitochondrial dysfunction in infectious diseases and how they relate to cancer pathogenesis.

Mitochondria are dynamic organelles that frequently change shape and distribution in response to stress or metabolic changes. Under severe stress (e.g., prolonged nutrient deprivation or electron transport chain inhibition), mitochondria undergo fission, while milder stressors (e.g., moderate starvation, UV exposure) trigger fusion [243]. Fission and fusion, regulated by proteins like dynamin-related protein 1 (DRP1), mitofusins (Mfn1/2), and optic atrophy protein 1 (OPA1), are essential for maintaining mitochondrial function, size, and shape [244]. Fission helps remove damaged mitochondria through mitophagy, while fusion supports content exchange, lipid metabolism, and DNA integrity [245]. Both processes are crucial for cell health, influencing mitochondrial positioning and interactions with other organelles. Obesity has been linked to increased mitochondrial fragmentation and insulin resistance, which has been associated with various diseases, including diabetes, cardiovascular diseases, neurodegenerative disorders, and cancer [246,247]. Additionally, gingipains, secreted by *P. gingivalis*, activate protease-activated receptor 2 (PAR-2), leading to, increased TNFα, MMP-9, and endothelial permeability, factors all implicated in cancer progression and mitochondrial cell death [248]. Elevated PAR-2 levels have been shown to contribute to increased cancer pathology, further linking mitochondrial dysfunction to cancer development [249,250,251,252,253].

**Figure 4:**
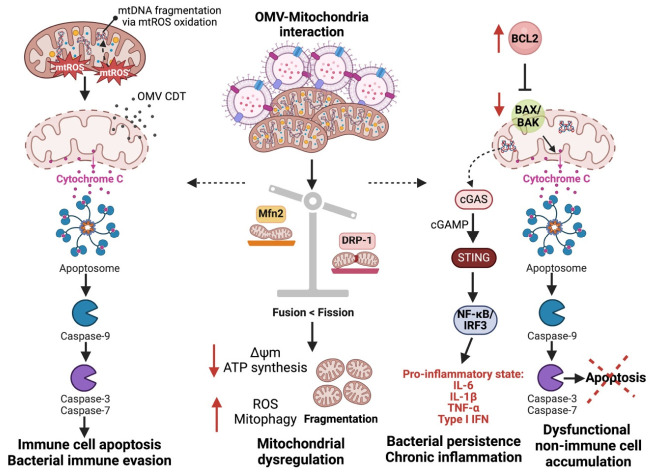
**Oral pathobionts (*P. gingivalis* and *F. nucleatum*) OMVs-Mitochondria crosstalk and its impact on mitochondrial dysfunction and cell fate.** OMVs manipulate mitochondrial dynamics to regulate cell survival, immune evasion, and inflammatory responses, contributing to chronic bacterial infection and disease progression. OMVs promote mitochondrial fission through upregulation of dynamin-related protein 1 (DRP1) and downregulation of mitofusin 2 (Mfn2), disrupting the balance between fusion and fission. This results in mitochondrial fragmentation, loss of membrane potential (Δψm), reduced ATP synthesis, and increased reactive oxygen species (ROS) production, leading to mitophagy and mitochondrial dysfunction. OMVs deliver virulence factors such as cytolethal distending toxin (CDT) that induce mitochondrial stress, leading to the release of cytochrome C, formation of apoptosome, and activation of caspase-9, 3, 7, driving immune cell apoptosis. This promotes bacterial immune evasion by reducing the host’s immune response. On the other hand, OMVs inhibit apoptosis in non-immune cells by upregulating anti-apoptotic protein BCL2 and downregulating pro-apoptotic proteins BAX/BAK. This prevents the release of cytochrome C, promoting cell survival and accumulation of dysfunctional cells. Mitochondrial dysfunction caused by OMVs leads to the release of mitochondrial DNA (mtDNA), which triggers the cGAS-STING pathway. This activates pro-inflammatory signalling through NF-ΚB and IRF3, leading to the production of cytokines such as IL (Interleukin)-6, IL-1*β*, TNF-*α*, and type I interferons, promoting bacterial persistence and chronic inflammation. BCL2: B-cell lymphoma 2, BAX: BCL2-associated X protein, BAK: BCL2 homologous antagonist killer), cGAS: Cyclic GMP-AMP synthase, NF-κB: Nuclear Factor kappa-light-chain-enhancer of activated B cells, IRF3: Interferon Regulatory Factor 3, TNF-*α*: Tumour Necrosis Factor alpha.

*P. gingivalis* OMVs can disrupt mitochondrial energy homeostasis through the delivery of virulence factors such as LPS that alter mitochondrial dynamics and reactive oxygen species signalling, influencing downstream apoptotic and inflammatory pathways (Figure 4). These OMVs have been implicated in inducing DRP1-dependent mitochondrial fission. Studies demonstrate that *P. gingivalis* LPS delivered via OMVs promotes upregulation of total DRP1 and phosphorylated DRP1 (Ser616), while reducing expression of the mitochondrial fusion protein Mfn2 in both mouse gingival tissues and human gingival fibroblasts. This shift toward enhanced mitochondrial fission results in fragmented and dysfunctional mitochondria [254]. Notably, knockdown of DRP1 reverses these effects, restoring normal mitochondrial morphology, complex I activity, and ATP production in HGFs exposed to *P. gingivalis* OMV [255]. In the context of cancer, excessive mitochondrial fission is increasingly recognized as a driver of metabolic reprogramming, ROS generation, and resistance to apoptosis. DRP1-mediated mitochondrial fragmentation has been associated with enhanced tumour cell proliferation, invasiveness, and adaptation to hypoxic stress. Therefore, OMV-induced mitochondrial dysfunction may promote a tumour-supportive metabolic phenotype, facilitating cancer cell survival, progression, and potential therapeutic resistance.

This mitochondrial dysfunction has significant implications for cancer progression. Excessive mitochondrial fission and fragmentation are known to promote metabolic reprogramming, favouring glycolysis over oxidative phosphorylation-an alteration commonly observed in cancer cells (known as the Warburg effect) [256]. Additionally, mitochondrial dysfunction can increase reactive oxygen species production, leading to DNA damage, genomic instability, and pro-survival signalling, all of which contribute to tumour initiation and progression [249]. Furthermore, DRP1 upregulation has been linked to enhanced cancer cell migration, invasion, and resistance to apoptosis [257]. Therefore, *P. gingivalis*-induced mitochondrial fragmentation may create a cellular environment that facilitates oncogenic transformation, potentially linking periodontal infection to increased cancer risk and progression. Mitochondrial reactive oxygen species (MitoROS) are known to play a key role in the immune response to bacterial infections [258]. Bacterial components like LPS and toxins, known as pathogen-associated molecular patterns, are recognized by host receptors such as Toll-like and Nod-like receptors (NLR) [259]. In macrophages, activation of TLRs stimulates MitoROS production and recruits mitochondria to phagosomes [259], while NLRX1 activation in the mitochondrial matrix also increases MitoROS [260]. MitoROS can directly damage pathogens and activate NADPH oxidases for further ROS production, enhancing bacterial killing [261,262]. However, excessive MitoROS also plays a critical role in cancer progression by promoting oxidative stress, DNA damage, and genomic instability-hallmarks of tumorigenesis [256]. In addition, MitoROS triggers inflammatory signalling through transcription factors like NF-κB and inflammasome assembly via NOD-like receptor thermal protein domain associated protein 3 (NLRP3) and NLRC4, creating a chronic inflammatory environment that supports cancer initiation and progression [263,264,265]. Persistent inflammation driven by MitoROS can enhance tumour cell survival, proliferation, and immune evasion, further linking bacterial infection and mitochondrial dysfunction to oncogenesis.

Mitochondria regulate immune responses through metabolic reprogramming [266]. During bacterial infection, macrophages undergo a shift from oxidative phosphorylation to aerobic glycolysis, promoting a pro-inflammatory phenotype. In response to LPS stimulation, key tricarboxylic acid (TCA) cycle enzymes such as isocitrate dehydrogenase 1 (IDH1) and aconitase are inhibited, leading to the accumulation of citrate and cis-aconitate [267,268]. Cis-aconitate is subsequently converted into itaconate by aconitate decarboxylase 1 (ACOD1, also known as IRG1). Itaconate exerts antimicrobial activity by inhibiting bacterial metabolic enzymes and disrupting pathogen survival pathways [269]. However, many pathogens have evolved mechanisms to degrade itaconate, enabling their persistence within macrophages [270].

Importantly, ACOD1-driven itaconate production has recently been linked to cancer immunopathology. Cancer cells and tumour-associated macrophages expressing ACOD1 have been shown to secrete T cell inhibitory factors that suppress CD8^+^ T cell activation and proliferation, thereby promoting immune evasion and tumour progression [271]. In the context of oral pathobionts, our previous work demonstrated that *P. gingivalis* OMVs increase macrophage inducible nitric oxide synthase (iNOS) expression via itaconate-dependent pathways [192]. This suggests that OMV-driven metabolic reprogramming of macrophages may contribute not only to antimicrobial responses but also to chronic inflammatory signalling within the tumour microenvironment.

Furthermore, *P. gingivalis* possesses adaptive mechanisms to withstand oxidative stress, including upregulation of antioxidant regulators such as OxyR and RprY [272], as well as the formation of a protective haem layer that shields against oxidative damage [273,274]. These antioxidant defences are further enhanced within plaque biofilms, supporting bacterial persistence [274,275]. Collectively, these findings indicate that *P. gingivalis* OMVs may drive immunometabolic rewiring of macrophages via ACOD1-itaconate signalling, while simultaneously resisting oxidative clearance, thereby fostering a tumour-permissive and immunosuppressive microenvironment.

Importantly, the reaction converting cis-aconitate to itaconate is catalyzed by the ACOD1 enzyme, which has been directly implicated in cancer pathology. Cancer cells that express ACOD1 secrete T cell inhibitory factors that suppress CD8^+^ T cell activation and proliferation, promoting an immunosuppressive tumour microenvironment [276]. It is observed that *P. gingivalis* OMVs increase macrophage iNOS via increased itaconate production [191]. Since iNOS-derived nitric oxide can contribute to chronic inflammation and DNA damage, this mechanism may further support an oncogenic environment. Collectively, these findings suggest that *P. gingivalis*-induced metabolic reprogramming in macrophages could not only enhance bacterial survival but also contribute to cancer progression by fostering inflammation and immune evasion.

OMVs have been shown to disrupt the dynamic processes of fusion and fission, leading to mitochondrial fragmentation and dysfunction. For instance, LPS-containing OMVs can impair mitochondrial fusion by inhibiting key fusion proteins such as mitofusins, resulting in the fragmentation of the mitochondrial network [277,278]. The interaction between OMVs and host cells can lead to their internalization through endocytosis, macropinocytosis, or direct fusion with the plasma membrane. Once internalized, OMVs can influence various intracellular processes. Recent studies suggest that some OMVs may directly interact with mitochondria, either through specific targeting mechanisms or indirectly by releasing factors that affect mitochondrial function [199].

Research has shown that certain bacterial toxins, such as cytolethal distending toxin (CDT) delivered via OMVs, can localize to the mitochondria, where they disrupt mitochondrial membrane integrity and the induction of mitochondrial permeability transition pores (MPTP), which result in the leakage of pro-apoptotic factors such as cytochrome c into the cytoplasm [279,280]. Released cytochrome c induces the formation of the apoptosome, which activates apoptotic caspase-9 and the downstream effector caspases (caspase-3 and -7), analogous to inflammasomes and the regulation of pyroptotic and apoptotic caspases [281,282]. Under normal conditions, pro-apoptotic proteins such as B cell lymphoma 2 (BCL-2)-like proteins, restraining pro-death BCL-2-associated x protein (BAX) and BCL-2 antagonist killer (BAK), translocate from the cytosol to the outer mitochondrial membrane (OMM) upon activation. This translocation triggers conformational changes and oligomerization that regulate the mitochondrial outer membrane permeability (MOMP), subsequently facilitating the release of key apoptotic mediators [283,284]. However, *P. gingivalis* appears to manipulate this process by downregulating the expression of the pro-apoptotic protein Bax while upregulating the anti-apoptotic protein BCL-2. This action promotes cell viability even after 24 h of infection, effectively maintaining cellular activity and preventing apoptosis [285]. This bacterial interference with apoptosis has significant implications for cancer progression. The suppression of BAX and upregulation of BCL-2 promote cell survival under conditions that would normally trigger programmed cell death, potentially enabling the accumulation of genetic mutations and prolonged cellular lifespan—both key factors in oncogenesis. Moreover, increased BCL-2 expression is a well-established mechanism of chemoresistance in various cancers, including oral squamous cell carcinoma and colorectal cancer [286]. By preventing apoptosis, *P. gingivalis* may contribute to a microenvironment that supports tumour initiation and resistance to therapy [287]. Chronic infections and inflammation induced by *P. gingivalis* further exacerbate these effects, fostering an environment conducive to malignant transformation and cancer progression [288].

It is reported that 60% of patients with chronic periodontitis exhibit various forms of mitochondrial structural abnormalities in their gingival tissue, suggesting that mitochondrial dysfunction caused by *P. gingivalis* may play a crucial role in the pathogenesis of periodontitis [289,290]. Studies have shown that *P. gingivalis* OMVs induce mitochondrial dysfunction by promoting the loss of mitochondrial membrane potential, increasing ROS production, and impairing ATP synthesis [291,292,293]. This mitochondrial stress can lead to the activation of mitophagy, a process where damaged mitochondria are selectively degraded [294]. Additionally, *P. gingivalis* OMVs may induce mitochondrial DNA (mtDNA) release into the cytosol, triggering the activation of the cyclic GMP-AMP synthase (cGAS)-stimulator of interferon genes (STING) pathway, which initiates a pro-inflammatory and type I interferon (IFN) response [290,295,296]. This mitochondrial-mediated inflammation contributes to the chronic inflammatory environment observed in periodontitis and may extend to systemic inflammation linked to cardiovascular diseases [297]. While these processes have been primarily studied in periodontitis, they are also key contributors to cancer progression. Chronic mitochondrial stress and mtDNA release are well-documented in tumour biology, where they promote inflammation, genomic instability, and immune evasion—hallmarks of cancer. The cGAS-STING pathway, in particular, has been implicated in tumorigenesis, as its activation can lead to either anti-tumour immune responses or, paradoxically, a tumour-promoting chronic inflammatory environment depending on the context [298]. Furthermore, excessive mitophagy in cancer cells can support tumour survival by selectively removing damaged mitochondria, preventing apoptosis, and adapting metabolic activity to favour tumour growth [299]. Increased ROS production, another consequence of *P. gingivalis*-induced mitochondrial dysfunction, can further drive DNA mutations, enhance cell proliferation, and activate oncogenic signalling pathways. Given these parallels, *P. gingivalis*-mediated mitochondrial dysfunction in periodontitis may not only contribute to local tissue destruction but could also enhance systemic inflammation and tumour progression in susceptible individuals.

One of the primary mechanisms through which *P. gingivalis* OMVs contribute to mitochondrial dysfunction is by promoting the loss of mitochondrial membrane potential (Δψm) [193]. The mitochondrial membrane potential is crucial for the proper functioning of mitochondria, as it is directly involved in ATP synthesis and maintaining overall mitochondrial health. Δψm is a critical regulator of mitochondrial bioenergetics, and its loss compromises cellular ATP production, severely impairing energy metabolism [300]. This disruption is particularly detrimental in cancer cells, where mitochondrial function plays a key role in supporting the high energetic demands associated with uncontrolled cell proliferation. In cancer, the loss of Δψm leads to a shift in energy metabolism, exacerbating the Warburg effect, in which cells rely more on glycolysis than oxidative phosphorylation, even in the presence of oxygen [301]. This metabolic shift, while providing some energy, is inefficient and increases the production of ROS, which in turn causes further mitochondrial damage and genetic instability [206]. The inability of cancer cells to generate sufficient ATP through oxidative phosphorylation, due to compromised Δψm, forces the cells to rely on alternative pathways such as glycolysis, contributing to the Warburg effect and enhancing tumorigenic potential.

Additionally, the loss of Δψm in cancer cells compromises the integrity of the mitochondrial inner membrane, which disrupts mitochondrial apoptosis regulation [302]. This is particularly concerning in cancer pathology, as the inability of mitochondria to induce cell death signals enables cancer cells to evade apoptosis, a crucial defence mechanism against tumorigenesis. As a result, mitochondrial dysfunction not only supports the energetic needs of rapidly dividing cancer cells but also facilitates tumour progression by increasing resistance to cell death and enhancing metastatic potential [303]. These cellular alterations make mitochondrial dysfunction a critical factor in the pathology of cancer, as it underpins both metabolic reprogramming and resistance to therapy. In summary, the crosstalk between *P. gingivalis* OMVs and mitochondria involves a complex interplay of cellular uptake mechanisms, mitochondrial dysfunction, oxidative stress, and alterations in cellular signalling pathways. This interaction of *P. gingivalis* OMVs and mitochondria highlights the role of OMVs in the pathogenesis of periodontal disease and underscores the importance of these OMVs may play in cancer pathology via mitochondria disruption and that targeting these mechanisms and OMVs are potential therapeutic interventions [292].

### 1.10. Engineered OMV-Based as Vehicle for Therapy

While *P. gingivalis* and some oral pathobionts’ OMVs contribute to cancer progression, it is important to recognize that not all OMVs are detrimental to cellular health or tumour biology. In fact, OMVs are a fundamental part of bacterial communication and have been shown to have a range of biological effects, including immune modulation, which could have beneficial or neutral roles in different contexts. For instance, OMVs derived from commensal bacteria can help maintain mucosal immunity and protect against infections [304]. In certain cases, OMVs can even stimulate immune responses that promote anti-tumour activity. OMVs from specific microbial communities have been implicated in enhancing the activity of immune cells, such as dendritic cells, which play a critical role in tumour surveillance and response [234]. Furthermore, OMVs from certain bacteria have been shown to induce an adaptive immune response that can potentially aid in controlling cancer growth [305]. Thus, while *P. gingivalis* OMVs are implicated in promoting mitochondrial dysfunction and cancer progression, OMVs, in general, can also have protective and immune-boosting effects depending on the bacterial species and the host environment.

## 2. Therapeutic Targeting of Oral Pathobionts’ OMVs: Opportunities and Challenges: OMVs in Cancer Immunotherapy and Vaccines

Immunotherapy has recently shown significant advantages in treating malignancies, complementing traditional methods such as surgery, radiation, chemotherapy, and targeted therapy. The most widely used immunotherapies include checkpoint inhibitors targeting PD-1/PD-L1 and CAR-T cell therapies, while others remain under clinical investigation [306]. Despite notable progress, concerns about the effectiveness and safety of immunotherapy limit its broader clinical application [307]. There is a critical need for novel approaches, including enhanced drug delivery and neoantigen presentation, to improve tumour treatment.

**Figure 5:**
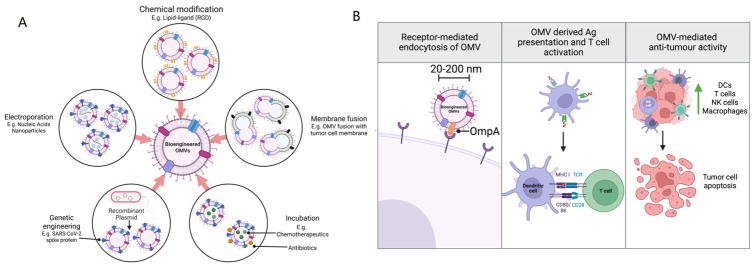
**Strategies for Bioengineering OMVs and their application in cancer immunotherapy.** (**A**) OMVs can be modified through several techniques: Chemical modifications, such as lipid ligands, e.g., Arginylglycylaspartic acid (RGD) tripeptide to improve targeting to cancer cells; Electroporation, allowing introduction of nucleic acids or nanoparticles into OMVs for enhanced therapeutic functions; Genetic engineering, by incorporating recombinant plasmids to express antigens, e.g., SARS-CoV-2 spike protein for vaccine development; Incubation with therapeutics, allowing loading of OMVs with chemotherapeutic agents or antibiotics, making them versatile delivery vehicles for range of drugs; Membrane fusion, allowing OMVs to fuse with tumour cell membranes for direct, targeted, and efficient method for delivering anti-cancer therapies. (**B**) Bioengineered OMVs are taken up by antigen-presenting cells (APCs) via receptor-mediated endocytosis, a process facilitated by OMV proteins, such outer membrane protein A (OmpA). Once internalized, OMVs deliver tumour antigens to dendritic cells (DCs), which process and present these tumour antigens (Ags) on MHC class I molecules to T cells. This interaction triggers the activation of cytotoxic T cells, enhancing the immune response against tumour cells. Bioengineered OMVs also stimulate the activation of other immune cells such as NK cells and macrophages, which collectively with DCs and T cells, lead to increased tumour cell apoptosis and effective anti-tumour immune response.

Various strategies have been reported for the application of bioengineered OMVs in cancer immunotherapy, as depicted in Figure 5. Recent research has explored the potential of bacterial-derived therapies in cancer treatment, particularly oral vaccines that stimulate cytotoxic T lymphocytes (CTLs) responses through tumour antigens. As the intestine has 70% of the body’s immune cells, oral vaccines could elicit strong anti-tumour responses. OMVs from commensal bacteria show promise in modulating the gut microbiota and immune responses, potentially enhancing immunotherapies for gastrointestinal (GI) tumours [236,308]. OMVs can utilize toxins as adhesins, enabling the vesicles to enter host cells via receptor-mediated endocytosis [45]. This entry mechanism is supported by common OMV components such as outer membrane protein A (OmpA), which, when present on the vesicle membrane, has been shown to fully activate macrophage cytokine production- an effect not replicated when OmpA is isolated from the vesicle membrane. Additionally, OMVs’ small size (20–200 nm) is critical for their effective processing by antigen-presenting cells (APCs) [309]. Their size, coupled with the ability to present a wide range of surface antigens in their native conformation, facilitates their entry into lymphatic vessels and subsequent uptake by APCs [42].

Studies have demonstrated that OMVs, such as those from *Akkermansia muciniphila*, can improve the efficacy of immune checkpoint blockade in GI cancers [310]. Over the last decade, engineered OMVs have shown promise in drug delivery and immunotherapy, yet few reviews have focused on their application in tumours [311]. This section addresses key engineering strategies, including genetic modification, drug loading, and surface modification, while discussing the advantages and limitations of OMVs in tumour treatment. We also explore the challenges of translating these emerging platforms into clinical practice. OMVs offer several advantages, including greater drug-loading capacity, stability, biocompatibility, and lower cytotoxicity. While they have intrinsic immune-boosting Bold formatting is necessaryproperties, their anti-tumour effects alone are limited, prompting the development of engineered OMVs for improved targeting, safety, and efficacy, particularly when combined with nanotechnology. These advancements offer promising avenues for more effective cancer therapies.

OMVs possess several inherent properties that make them ideal candidates for vaccine development [312]. OMVs, particularly those derived from Gram-negative bacteria, exhibit strong immunogenicity, function as self-adjuvants, and are naturally taken up by immune cells, making them valuable in vaccine development against pathogenic bacteria. Unlike live bacteria, OMVs are inanimate, making them safer and moderately reactogenic, which led to their early proposal as a vaccine platform against bacterial pathogens. With advancements in genetic engineering and nanotechnology, OMVs have become promising vaccine candidates, offering benefits in safety, cost-effectiveness, and scalability.

One of the most significant breakthroughs in OMV-based vaccines is the development of the group B meningococcal vaccine. The first OMV-based vaccine, known as the Cuban vaccine, was derived from group B *Neisseria meningitidis* and provided long-lasting, bactericidal antibody responses against meningococcal disease [313,314]. Further advancements led to the approval of the multi-component vaccine 4CMenB by both the European Medicines Agency (EMA) and the US Food and Drug Administration (FDA). 4CMenB combines OMVs from group B *Meningococci* with recombinant antigens, effectively triggering strong immune responses across various age groups, including adults, adolescents, and infants [315]. Beyond meningococcal vaccines, significant progress has been made in developing OMV-based vaccines for other pathogens. For instance, OMVs loaded with the O-antigen polysaccharide of *Shigella sonnei* have shown promise in protecting against bacillary dysentery [316]. Tian et al. developed an OMV vaccine based on recombinant *Salmonella* expressing *S. sonnei* O-antigen via the LPS synthesis pathway [317]. Additionally, encapsulating OMVs in polyanhydride nanoparticles has shown protective effects in mouse models against *Shigella* infection via nasal or oral immunization [318].

Moreover, OMVs are being explored for protection against viral infections. For example, OMVs have been engineered to display the receptor-binding domain (RBD) of the SARS-CoV-2 spike protein, targeting viral entry through angiotensin-converting enzyme 2 (ACE2) receptors [319]. Zhang et al. developed hybrid membrane vesicles (HMVs) by fusing eukaryotic membrane vesicles expressing the spike protein with bacterial OMVs, enhancing antiviral immune responses [319]. Similarly, Yang et al. engineered OMVs displaying the RBD on their surface, which, when injected into mice, accumulated in lymph nodes and induced strong SARS-CoV-2-specific immune responses, showcasing the potential of OMVs as a platform for viral vaccines [320]. In summary, OMVs have shown great promise as a versatile and effective platform for developing vaccines against both bacterial and viral infections, with several key advancements highlighting their potential in preventing epidemic diseases.

Additionally, their inherent immunogenic properties, coupled with the ability to be engineered for specific therapeutic purposes, position OMVs as versatile tools in oncology [217]. OMVs, by virtue of their bacterial origin, inherently contain PAMPs that are recognized by the immune system via TLRs [321]. This recognition triggers robust immune responses, activating both the innate and adaptive immune systems. For example, upon LPS release from OMVs, it binds to CD14 on host cells and is recognized by the TLR4-MD2 complex, triggering an inflammatory response via TLR4 dimerization [322]. OMVs from bacteria such as *E. coli*, *P. aeruginosa*, and *N. meningitidis* activate this pathway effectively. For instance, OMVs from *P. aeruginosa* activate the MyD88-dependent NF-κB signalling pathway, while OMVs from *H. pylori* initiate the MAPK/AP-1 signalling cascade [323]. Additionally, OMVs from pathogens like *P. aeruginosa*, *H. pylori* and *Neisseria gonorrhoeae* can activate inflammasome signalling in macrophages, leading to the release of inflammatory cytokines and inducing pyroptosis [191,240,324].

Interestingly, not all OMVs induce pro-inflammatory effects. Symbiotic bacteria like *Bacteroides fragilis* release OMVs containing capsular polysaccharide A (PSA), which exerts anti-inflammatory effects by interacting with TLR2 on dendritic cells, mediated by the protein Gadd45α [180]. Some OMVs activate multiple host pattern recognition receptors simultaneously. For example, OMVs from *M. catarrhalis* stimulate host B cells via both TLR2 and TLR9 [325], while OMVs from enterohemorrhagic *Escherichia coli* (EHEC) O157 induce IL-8 production through TLR4 and TLR5 signalling [326]. Notably, OMVs may activate different pathways compared to their parent bacteria due to the absence of certain components, such as bacterial ribosomal RNA, which TLR13 recognizes in bacteria but not in OMVs [327]. Host pattern recognition receptors signalling pathways play a key role in OMV-mediated anti-tumour activity, leading to the activation of dendritic cells, macrophages, and NK cells. The activation of these immune cells facilitates the presentation of tumour antigens to CTLs, a critical step in mounting an effective anti-tumour immune response [328].

### 2.1. OMVs for Drug Delivery Platforms

Alongside their inherent immunogenic properties, OMVs are emerging as a promising platform for drug delivery in cancer immunotherapy. OMVs are highly resistant to enzymatic degradation, allowing them to travel long distances and reach target sites effectively. Their rapid detection and uptake by host cells have made them valuable as delivery vehicles for various biomedical applications. Several methods are used to load substances into OMVs, including genetic engineering, incubation, electroporation, membrane fusion, and chemical modifications [329]. Genetic engineering is the most common method, enabling the loading of specific proteins into OMVs by introducing plasmids into the parent bacteria. For example, native lipoproteins like SlyB can be used as anchor points to display cargo on the OMV surface [330]. Advanced systems such as SpyCatcher/Tag [331,332] and ClyA fusion proteins [333] allow OMVs to display functional molecules, enhancing their targeting ability. Incubation methods involve loading chemotherapeutic drugs or dyes into OMVs by directly mixing them with either OMVs or parent bacteria, which can sort the substances into OMVs [334,335]. This technique is particularly useful for antibiotic loading, though it may be less effective for certain drugs due to bacterial efflux mechanisms [336].

Electroporation, which uses high-voltage pulses to create membrane pores, has also been employed to load nucleic acids and nanoparticles into OMVs without compromising their integrity [337]. Other techniques, such as membrane fusion, sonication, and physical extrusion, enable OMVs to introduce external antigens and develop new functionalities, although these methods may sometimes disrupt membrane integrity, leading to severe leakage of cytoplasmic contents in OMVs [338,339]. Chemical modifications of OMVs, leveraging their lipid and protein content, offer additional opportunities to anchor cargo molecules, enhancing their functionality [334]. For instance, OMVs can be decorated with targeting ligands like Arginylglycylaspartic acid (RGD) peptides or conjugated with molecules like calcium phosphate for specific applications [340]. Overall, these diverse loading strategies for OMVs have paved the way for their use in various biomedical applications, from drug delivery to immune modulation.

One of the primary advantages of OMVs in anti-tumour therapy is their modifiability [164]. Engineered OMVs can be tailored to express tumour-specific antigens or immune-modulating molecules, thus enhancing their specificity and efficacy in tumour targeting. For instance, by decorating OMVs with tumour-associated antigens (TAAs) such as carcinoembryonic antigen (CEA) or mutant KRAS proteins, these vesicles can be used to effectively prime the immune system to recognize and eliminate tumour cells harbouring these mutations [217]. This targeted approach holds great promise in treating gastrointestinal (GI) tumours, which often harbour unique mutational profiles and present challenges for conventional immunotherapies.

Gao et al. engineered *E. coli* to express TNF-related apoptosis-inducing ligand (TRAIL) protein and modified the resulting OMVs with an αvβ3 integrin peptide, targeting ligand, and indocyanine green for a more targeted therapeutic approach against melanoma [335]. These multifunctional OMVs enabled transdermal, photo-activated TRAIL-mediated therapy, enhancing the anti-tumour effects. Similarly, Tang et al. modified OMVs derived from attenuated *Salmonella* with the tumour-targeting Arg-Gly-Asp (RGD) peptide to boost their tumour-targeting ability [334]. These OMVs were then used to coat polymeric micelles loaded with chemotherapeutic agents, greatly enhancing the synergistic effects of immunotherapy and chemotherapy. Systemic administration of these OMVs not only elicited a strong immune response to prevent melanoma development but also significantly inhibited tumour growth and metastasis, thereby extending the survival rates of tumour-bearing mice.

In other studies, Chen et al. developed a complex of gold nanoparticles and OMVs derived from *E. coli*, demonstrating that combining this complex with radiotherapy substantially increased intracellular ROS, leading to enhanced radiosensitization and immune activation, and successfully inhibiting tumour growth in glioblastoma models [341]. Additionally, research on OMVs derived from attenuated *Klebsiella pneumoniae* demonstrated their effectiveness when combined with the chemotherapeutic agent doxorubicin (DOX), inducing an immune response against lung cancer without significant toxicity in vivo [342].

The potential of OMVs for cancer treatment has been further highlighted by Kim et al., who showed that OMVs derived from *E. coli* W3110 could accumulate in tumour tissues and trigger an IFNγ-dependent antitumour response [343]. However, despite their ability to initiate strong IFNγ-mediated immune responses, IFNγ paradoxically upregulates immunosuppressive factors like programmed death 1 ligand 1 (PD-L1) in the tumour microenvironment, which inhibits T cell function and reduces immunotherapy efficacy. To address this, Li et al. genetically engineered *E. coli* to express the PD-1 ectodomain on the OMV surface. These OMVs were able to bind PD-L1 on tumour cells, leading to delayed tumour growth in melanoma and colon cancer mouse models by combining immune stimulation with checkpoint inhibition [344]. Building on this concept, Pan et al. developed OMVs modified with the tumour-targeting peptide Lyp1 and introduced PD-1 plasmids via electroporation [345]. Lyp1 facilitated OMV entry into tumour cells, where the PD-1 plasmid was delivered to the nucleus, leading to overexpression of PD-1 on tumour cells. This allowed PD-1 to bind PD-L1, blocking the PD-1/PD-L1 pathway and preventing immune escape. At the same time, these OMVs recruited cytotoxic T lymphocytes and natural killer cells, inducing IFNγ secretion and amplifying the antitumor immune response.

The immunosuppressive TME, which fosters immune evasion and reduces the effectiveness of immunotherapies, has been a significant obstacle in cancer treatment. To overcome this, Qing et al. developed OMVs coated with a pH-sensitive calcium phosphate (CaP) shell [340]. This CaP coating shields OMVs from clearance and reduces their toxicity, while simultaneously neutralizing the acidic TME and promoting the polarization of macrophages toward the pro-inflammatory M1 phenotype, enhancing the therapeutic efficacy of OMVs in cancer immunotherapy.

### 2.2. Targeting OMV Biogenesis and Function

Beyond their application as therapeutic delivery platforms, OMVs themselves represent promising therapeutic targets, particularly in the context of tumour-promoting oral pathobionts. Inhibiting OMV biogenesis may reduce the release and dissemination of virulence factors, thereby limiting OMV-mediated inflammation, immune modulation, and oncogenic signalling. OMV formation in Gram-negative bacteria is a tightly regulated process influenced by outer membrane composition, peptidoglycan–lipoprotein interactions, and environmental stress responses. Disruption of these processes has been shown to significantly alter OMV production. For example, perturbations in the linkage between the outer membrane and the peptidoglycan layer, such as alterations in Braun’s lipoprotein, can enhance or suppress vesicle formation [42]. Similarly, accumulation of misfolded proteins in the periplasm and envelope stress responses, including activation of the σ^E pathway, have been implicated in increased OMV production [43]. Targeting OMV-associated virulence factors represents another potential strategy. In the case of *P. gingivalis*, inhibition of gingipains has been proposed as a therapeutic approach to limit OMV-mediated proteolysis, immune dysregulation, and tumour-promoting inflammation [346]. Likewise, blocking key adhesins such as FadA and Fap2 in *Fusobacterium nucleatum* may reduce OMV-mediated tumour cell adhesion, invasion, and immune evasion [58,171].

Neutralization strategies directly targeting OMVs have also been explored. Antibodies against OMV components, including LPS and outer membrane proteins, may block OMV–host cell interactions and attenuate downstream signalling pathways [347]. In addition, modulation of the microbiome through antimicrobial therapies, probiotics, or targeted microbiota interventions may reduce the abundance of OMV-producing pathobionts and thereby indirectly limit OMV-mediated pathological effects. Although these strategies remain largely preclinical, they highlight the therapeutic potential of targeting OMV biogenesis and function as a novel approach to disrupt host–microbe interactions in cancer. Current and emerging therapeutic strategies targeting or utilizing bacterial OMVs in cancer are summarized in Table 3. Future research should prioritize the direct isolation and characterization of OMVs from patient-derived samples to better define their clinical relevance. In addition, understanding OMV heterogeneity and identifying distinct vesicle subpopulations with specific oncogenic or immunomodulatory functions will be critical. Addressing these gaps will be essential for translating OMV-based diagnostics and therapeutics into clinical applications. While these approaches show considerable promise, several challenges remain, as discussed below.

### 2.3. Challenges and Future Perspectives

Despite the promising therapeutic potential of OMV-based approaches, several critical challenges must be addressed before their translation into clinical practice. One of the primary concerns is the intrinsic immunogenicity of bacterial-derived OMVs. While this property underpins their utility as vaccine adjuvants and immunotherapeutic agents, excessive activation of the immune system may lead to systemic inflammation, toxicity, or unintended immune dysregulation [168]. Another major limitation is the lack of tumour-specific targeting. Following systemic administration, OMVs are rapidly recognized and cleared by the reticuloendothelial system, particularly by macrophages in the liver and spleen, which significantly reduces their bioavailability at tumour sites [176]. Although surface engineering strategies, such as ligand conjugation and nanoparticle hybridisation, have improved targeting efficiency, achieving precise and controlled delivery remains a significant challenge.

OMV heterogeneity represents an additional barrier to clinical application. OMVs are not uniform populations, rather, they exhibit variability in size, composition, and cargo depending on bacterial strain, growth conditions, and environmental stressors [347]. This heterogeneity complicates standardization, reproducibility, and quality control, which are essential for regulatory approval and large-scale clinical use.

Furthermore, scalable production and purification of OMVs remain technically challenging. Current isolation methods, including ultracentrifugation and density gradient separation, are labour-intensive and may not be suitable for industrial-scale manufacturing. The development of robust, standardized, and cost-effective production pipelines is therefore essential for clinical translation. Importantly, emerging evidence suggests that OMVs derived from oral pathobionts may exert dual roles in cancer, with both tumour-promoting and therapeutic effects depending on their origin and cargo. For example, OMVs from *Porphyromonas gingivalis* have been associated with immune evasion, metabolic reprogramming, and enhanced tumour cell plasticity. These findings highlight the need for careful design of OMV-based therapeutics to minimize potential pro-tumorigenic effects and ensure safety.

Future research should focus on improving the safety profile of OMVs through detoxification strategies (e.g., modification of LPS), enhancing targeting specificity via advanced bioengineering approaches, and integrating OMV-based therapies with existing immunotherapies such as checkpoint inhibitors. In addition, the identification of OMV-specific biomarkers may enable patient stratification and personalized therapeutic approaches. Overall, while OMV-based strategies remain in early stages of development, advances in synthetic biology, nanotechnology, and microbiome research are expected to accelerate their translation into clinically viable cancer therapies.

In conclusion, the versatility of OMVs in antigen presentation, immune modulation, and drug delivery, together with their capacity to overcome immune evasion mechanisms, positions them as promising tools in the next generation of cancer therapies. However, despite encouraging preclinical findings, significant challenges remain in translating OMV-based approaches into clinical practice [217]. Safety concerns regarding the administration of bacterial-derived components in humans must be addressed, particularly the risk of inducing excessive inflammation, immune dysregulation, or autoimmune reactions. Moreover, scalable production methods and standardization of OMV preparations are essential for the successful clinical translation of these therapies.

Importantly, growing evidence demonstrates that certain OMVs, particularly those derived from oral pathobionts such as *P. gingivalis*, can promote tumour progression through immune modulation, metabolic reprogramming, and enhancement of tumour cell plasticity. Therefore, future therapeutic strategies must be developed with careful consideration of these tumour-promoting mechanisms. A detailed mechanistic understanding of how OMVs interact with tumour cells and the tumour microenvironment will be critical to ensure that engineered OMV-based platforms are designed to minimize oncogenic potential and avoid inadvertently enhancing cancer development or progression.

## 3. Conclusions

Outer membrane vesicles (OMVs) are nanoscale, spherical structures naturally released by Gram-negative bacteria and play critical roles in intercellular communication, immune modulation, and pathogenesis. In the context of cancer, particularly malignancies associated with oral dysbiosis, OMVs derived from pathobionts such as *Porphyromonas gingivalis* and *Fusobacterium nucleatum* have emerged as key mediators linking chronic infection to tumour development and progression. These vesicles transport bioactive cargo, including lipopolysaccharide, proteases, adhesins, and nucleic acids, that can directly reprogram host cell signalling pathways, activate pro-inflammatory transcription factors (e.g., NF-κB, STAT3), promote immune checkpoint upregulation (PD-L1), induce epithelial–mesenchymal transition, and alter mitochondrial dynamics and immunometabolic responses.

Through these mechanisms, OMVs contribute to tumour cell plasticity, immune evasion, metabolic reprogramming, and the establishment of a tumour-permissive microenvironment. Importantly, while engineered OMVs hold promise as platforms for cancer immunotherapy, drug delivery, and vaccine development, naturally derived OMVs from oral pathobionts may exert tumour-promoting effects. Therefore, a comprehensive understanding of OMV–host interactions within the tumour microenvironment is essential to distinguish therapeutic potential from pathogenic risk. Future research must carefully evaluate both the oncogenic and immunomodulatory properties of OMVs to ensure that translational strategies harness their benefits without inadvertently promoting cancer progression.

## Figures and Tables

**Table 1:** Comparative features of outer membrane vesicles (OMVs) derived from major oral pathobionts implicated in cancer.

Feature	*Porphyromonas gingivalis* OMVs	*Fusobacterium nucleatum* OMVs
**Major OMV cargo**	Gingipains (RgpA, RgpB, Kgp), LPS, fimbriae (FimA, Mfa1), peptidoglycan, small RNAs [42,165,168]	FadA adhesin, Fap2 protein, LPS, outer membrane proteins (FomA), small RNAs [42,125]
**Dominant pattern recognition receptors**	Primarily TLR2 (also TLR4) [169]	Predominantly TLR4 [170]
**Key signalling pathways activated**	NF-κB, MAPK, PI3K/Akt, JAK/STAT3 [166,167]	NF-κB, β-catenin, TLR4-dependent pathways [58,125]
**Immune modulation**	Upregulation of PD-L1, induction of Tregs, impaired dendritic cell maturation [167]	Inhibition of NK cells and T cells via TIGIT (Fap2), M2 macrophage polarization [171]
**Effects on apoptosis**	Anti-apoptotic (increase BCL2/BAX ratio, BAD phosphorylation) [128]	Promotes survival via immune evasion and inflammatory signalling [125]
**Effects on DNA damage/genomic instability**	ROS-mediated DNA damage, impaired DNA repair, epigenetic modifications [132,172]	Impairment of mismatch repair (MMR), association with microsatellite instability [173]
**Epithelial–mesenchymal transition (EMT)**	Induces EMT via inflammatory and signalling pathways [174,175]	Strong EMT induction via FadA–E-cadherin–β-catenin signalling [58,176]
**Tumour microenvironment modulation**	Chronic inflammation, CAF activation, angiogenesis (VEGF, HIF-1α) [177]	Recruitment of MDSCs, macrophage polarization, pro-inflammatory niche formation [125]
**Cancer types most associated**	Oral squamous cell carcinoma, pancreatic cancer, gastric cancer [83,92]	Colorectal cancer, esophageal cancer, head and neck cancers [178]
**Unique distinguishing feature**	Gingipain-mediated proteolysis and immune checkpoint modulation [165,167]	Fap2-mediated immune evasion via TIGIT and selective tumour targeting [171]

**Table 2:** Summary of evidence linking oral pathobionts’ OMVs to cancer-related mechanisms.

Oral Bacterium	OMV Cargo/Mechanism	Model System	Cancer Type	Observed Phenotype	Evidence Strength	Limitations
***P. gingivalis***	Gingipains, LPS → NF-κB activation [165,166]	In vitro (epithelial cells)	Oral cancer	Increased inflammation, proliferation	Moderate (preclinical)	Limited clinical validation
***P. gingivalis***	OMV-induced PD-L1 expression [167,213]	In vitro, animal models	Gastric, prostate cancer	Immune evasion	Moderate	Mechanistic, not clinical
***P. gingivalis***	ROS induction, mitochondrial dysfunction [214]	In vitro	Multiple cancers	DNA damage, metabolic reprogramming	Moderate	Indirect evidence
***F. nucleatum***	FadA–E-cadherin → β-catenin activation [58]	In vitro, animal models	Colorectal cancer	Increased proliferation, EMT	Strong (well-established pathway)	OMV-specific contribution still emerging
***F. nucleatum***	Fap2–TIGIT interaction [171]	In vitro, in vivo	Colorectal cancer	NK/T cell inhibition	Strong	Mostly preclinical
***F. nucleatum***	OMV-induced TLR4 activation [170,176]	In vitro (HT-29 cells)	Colorectal cancer	↑ IL-8, TNFα, inflammation	Moderate	Limited in vivo validation
***P. gingivalis*** **&** ***F. nucleatum***	OMV-induced EMT and invasion [176,215]	In vitro	Oral, colorectal cancer	Migration, invasion, metastasis	Moderate	Model-dependent
**Mixed oral microbiome**	OMV-associated inflammatory signalling [125,216]	Clinical association studies	Multiple cancers	Dysbiosis linked to poor prognosis	Weak–moderate	OMVs not directly measured
**Engineered OMVs**	Drug delivery/immunotherapy [217,218,219]	Preclinical models	Various	Tumour targeting, immune activation	Emerging	No clinical trials yet

→ = causes; ↑ = increase.

**Table 3:** Therapeutic strategies targeting or utilizing bacterial OMVs in cancer.

Strategy	Approach	Mechanism	Status	Challenges
**Engineered OMVs** [348]	OMVs loaded with drugs/antigens	Targeted delivery, immune activation	Preclinical	Target specificity, safety
**OMV-based vaccines** [45,168,349]	Immunogenic OMVs	Stimulate anti-tumour immunity	Preclinical	Immunogenicity control
**Inhibition of OMV biogenesis** [42]	Blocking vesicle formation pathways	Reduce delivery of virulence factors	Experimental	Limited targeting strategies
**Neutralization strategies** [43]	Anti-OMV antibodies	Block OMV-host interaction	Conceptual/preclinical	Specificity, delivery
**Microbiome modulation** [158,350]	Reducing pathobionts	Decrease OMV production	Emerging	Complexity of microbiota

## Data Availability

No new data were created or analyzed in this study. Data sharing is not applicable to this article.
